# KRAS^G12D^ inhibition reprograms the tumor-induced immunosuppressive environment and enhances NK cell–mediated antitumor immunity

**DOI:** 10.1126/sciadv.aec9236

**Published:** 2026-07-23

**Authors:** Tuo Hu, Taiwei Mo, Lin Wang, Qiuqing Ke, Liangjie Chi, Hongyuan Chen, Chao Xu, Chenshen Huang, Aiping Lin, Shaoxin Cai, Haiyu Chen, Wenyu Wang, Longyang Jin, Junzhang Zhao, Haoyun Liu, Chuanyuan Liu, Yimamu Baihetiyaer, Abudumaimaitijiang Tuersun, Abuduhalike Abulimiti, Yan Zheng, Zuwei Wang, Shi Chen, Jie Cao, Shiyun Lu, Xueping Huang, Fangqin Xue, Chunbo He

**Affiliations:** ^1^ Department of General Surgery (Gastric Surgery, Colorectal Surgery), The Sixth Affiliated Hospital, Sun Yat-sen University, Guangzhou, Guangdong Province 510507, China.; ^2^ Guangdong Provincial Key Laboratory of Colorectal and Pelvic Floor Diseases, Guangdong Institute of Gastroenterology, The Sixth Affiliated Hospital, Sun Yat-sen University, Guangzhou, Guangdong Province 510507, China.; ^3^ Biomedical Innovation Center, The Sixth Affiliated Hospital, Sun Yat-sen University, Guangzhou, Guangdong Province 510507, China.; ^4^ Department of General Surgery, The First Affiliated Hospital of Jinan University, Jinan University, Guangzhou, Guangdong Province 510632, China.; ^5^ Gastroenterology Center, Fujian Provincial Hospital, Fuzhou University Affiliated Provincial Hospital, Fuzhou, Fujian Province 350001, China.; ^6^ Department of Microbiology and Immunology, Oklahoma Medical Research Foundation, Oklahoma City, OK 73104, USA.; ^7^ Department of Cardiovascular Surgery, Fujian Provincial Hospital, Fuzhou University Affiliated Provincial Hospital, Fuzhou, Fujian Province 350001, China.; ^8^ Department of Physiology, Oklahoma Medical Research Foundation, Oklahoma City, OK 73104, USA.; ^9^ Department of Oncology Science, University of Oklahoma Health Sciences Center, Oklahoma City, OK 73104, USA.; ^10^ Department of General Surgery, The Ganzhou People’s Hospital, Ganzhou, Jiangxi Province 341000, China.; ^11^ Department of General Surgery, The First People’s Hospital of Kashgar Region, Kashgar, Xinjiang Province 844001, China.; ^12^ Department of General Surgery, Kashi Prefecture Second People’s Hospital, Kashgar, Xinjiang Province 844001, China.; ^13^ Department of Oncology, Fujian Provincial Hospital, Fuzhou University Affiliated Provincial Hospital, Fuzhou, Fujian Province 350001, China.; ^14^ Department of Hepatobiliary Pancreatic Surgery, Fujian Provincial Hospital, Fuzhou University Affiliated Provincial Hospital, Fuzhou, Fujian Province 350001, China.; ^15^ Department of General Surgery, Guangzhou First People’s Hospital, South China University of Technology, Guangzhou, Guangdong Province 510180, China.; ^16^ Fujian Provincial Institute of Gastroenterology, Fujian Provincial Hospital, Fuzhou University Affiliated Provincial Hospital, Fuzhou, Fujian Province 350001, China.; ^17^ Institute of Precision Medicine, Fujian Provincial Hospital, Fuzhou University Affiliated Provincial Hospital, Fuzhou, Fujian Province 350001, China.

## Abstract

KRAS^G12D^ mutation drives oncogenic progression and creates an immunosuppressive microenvironment in cancers like pancreatic ductal adenocarcinoma and colorectal cancer. We investigate the immunomodulatory mechanisms of the KRAS^G12D^ inhibition and its synergy with natural killer (NK) cell therapies. We demonstrate that KRAS^G12D^ inhibition with MRTX1133 remodels the immune landscape by reducing myeloid-derived suppressor cell (MDSC) accumulation and facilitating infiltration and activation of NK and CD8^+^ T cells. Crucially, MRTX1133 reverses systemic immunosuppression, restoring the fitness of adoptively transferred NK cells. Mechanistically, KRAS^G12D^ inhibition impairs IFNGR1 palmitoylation and subsequent lysosomal degradation. MRTX1133 stabilizes IFNGR1 by reducing palmitoyltransferase expression and the palmitate pool. This stabilization increases IFN-γ/IFNGR signaling and up-regulates NK cell–activating ligands ICAM1 and ULBP1, thereby sensitizing cancer cells to NK cells. Consequently, combining MRTX1133 with IL-15 or adoptive NK cell therapy yields synergistic antitumor responses and prolonged survival. Our findings provide mechanistic rationale for combining KRAS^G12D^ inhibitors with NK cell–based immunotherapies to improve outcomes for patients with KRAS^G12D^-mutant cancers.

## Teaser

KRAS^G12D^ inhibition remodels the immune environment to favor NK cell activity and sensitizes cancer cells to NK cell killing.

## INTRODUCTION

Mutations in the *KRAS* gene are among the most prevalent oncogenic drivers in human cancers, with the KRAS^G12D^ variant being particularly frequent in aggressive malignancies such as pancreatic ductal adenocarcinoma (PDAC) and colorectal cancer (CRC) (*1*–*3*). The recent development of direct KRAS^G12D^ inhibitors, including MRTX1133 and RMC-9805, represents a substantial therapeutic advancement. These agents primarily function by suppressing the canonical RAF (Rapidly Accelerated Fibrosarcoma)–MEK (MAPK/ERK Kinase) signaling pathway (*2*, *4*). However, the full spectrum of mechanisms contributing to their clinical efficacy, particularly their influence on the tumor microenvironment (TME), remains to be fully elucidated.

The KRAS^G12D^ oncoprotein orchestrates tumor progression not only through cell-intrinsic mechanisms, such as metabolic reprogramming and the enhancement of cancer stemness (*5*, *6*), but also by extensively remodeling the TME. This includes the establishment of an immunosuppressive niche characterized by the secretion of protumor factors and the recruitment of suppressive immune cells (*7*). For instance, KRAS^G12D^-mutant PDAC cells secrete cytokines like granulocyte-macrophage colony-stimulating factor (GM-CSF) and interleukin-6 (IL-6), which recruit myeloid-derived suppressor cells (MDSCs) that inhibit cytotoxic T lymphocyte activity (*8*). Accordingly, treatment with the KRAS^G12D^ inhibitor MRTX1133 has been shown to reduce MDSC infiltration and enhance CD8^+^ T cell–mediated cytotoxicity (*9*). These findings underscore the profound impact of KRAS^G12D^ signaling on shaping an immune-evasive environment and suggest that targeting this pathway may synergize with immunotherapy.

Despite these insights, immunotherapies such as immune checkpoint blockade have shown limited success in PDAC and microsatellite-stable CRC (*10*, *11*). This is largely attributed to their “cold” TME, which lacks sufficient T cell infiltration and function (*12*, *13*). While considerable research has focused on the interplay between KRAS mutations and adaptive immunity, the connection with innate immune effectors is less understood. Natural killer (NK) cells are a critical component of the innate immune system, capable of eliminating malignant cells without prior sensitization and modulating adaptive immune responses (*14*). NK cell–based immunotherapies are emerging as a promising therapeutic modality for various cancers (*15*). However, the efficacy of these therapies in tumors like PDAC and CRC is often hampered by the low density and functional impairment of NK cells within the hostile TME (*16*, *17*).

Given that KRAS^G12D^ inhibition can modulate the immunosuppressive landscape (*9*, *18*–*20*), there is a strong rationale to investigate whether this approach could also restore the antitumor activity of NK cells. Here, we demonstrate that the KRAS^G12D^ inhibitor MRTX1133 enhances the susceptibility of cancer cells to NK cell–mediated killing. Mechanistically, we found that MRTX1133 treatment up-regulates the expression of NK cell–activating ligands, including intercellular adhesion molecules (ICAMs) and UL16-binding proteins (ULBPs), on tumor cells. Concurrently, it suppresses the secretion of immunosuppressive cytokines such as IL-6 and GM-CSF, which are known to promote MDSC proliferation. This dual action reshapes the TME to be more permissive to NK cell activity. Our findings reveal a mechanism by which targeted inhibition of KRAS^G12D^ bridges oncogenic signaling with innate immune regulation, providing a compelling basis for combining KRAS^G12D^ inhibitors with NK cell–based immunotherapies to treat these challenging malignancies.

## RESULTS

### KRAS^G12D^ inhibition remodels TIME and enhances NK cell activation

**Fig. 1.**
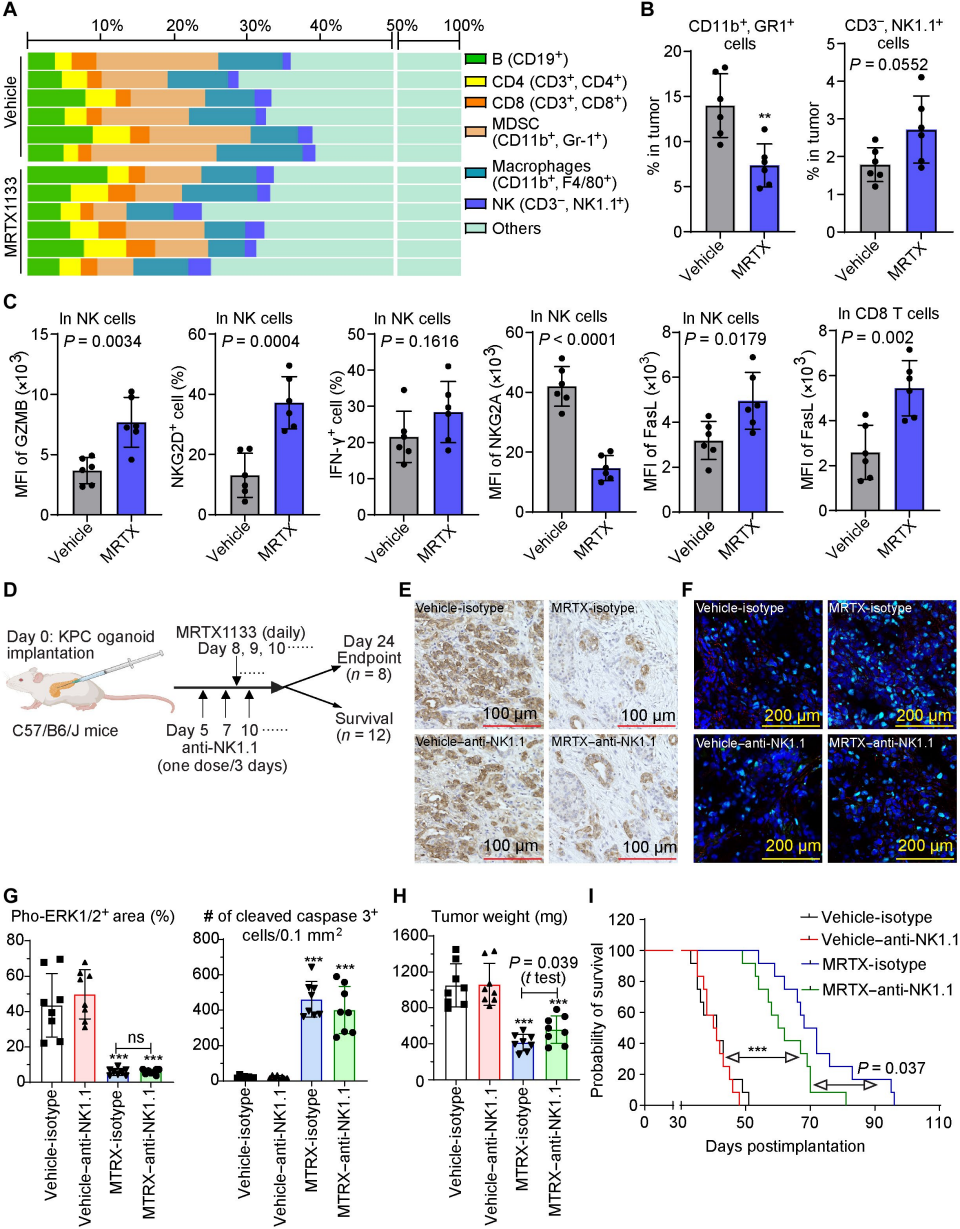
KRAS^G12D^ inhibition reprograms TME and enhances NK cell activation. (**A**) Flow cytometry analysis showing the relative abundance of indicated immune cell subsets in orthotopic KPC PDAC tumors from C57BL/6J mice treated with vehicle or MRTX1133 for 2 weeks (24 days postimplantation). (**B**) Quantification of MDSCs and NK cells as a percentage of total immune cells in orthotopic KPC tumors from the indicated treatment groups. (**C**) Flow cytometry analysis of IFN-γ, GZMB, NKG2D, NKG2A, and FASL expression in tumor-infiltrating NK cells and FASL expression in tumor-infiltrating CD8 T cells from orthotopic KPC tumors. MFI, median fluorescence intensity. (**D**) Schematic of experimental design for assessing the effect of NK cell depletion on MRTX1133 treatment efficacy in the orthotopic KPC tumor model. (**E** to **G**) Representative immunohistochemistry (IHC) and immunofluorescence (IF) images and corresponding statistical analyses of phosphorylated ERK1/2 (p-ERK1/2) and cleaved caspase-3 in orthotopic KPC tumors from vehicle- and MRTX1133-treated groups, with or without NK cell depletion. (**H**) Statistical analysis of tumor weight from orthotopic KPC tumors treated with vehicle or MRTX1133 following NK cell depletion. (**I**) Kaplan-Meier survival curves of orthotopic KPC tumor–bearing mice treated as indicated. Data are expressed as mean ± SEM. Statistical significance was determined using a two-tailed Student’s *t* test for (B) and (C) and one-way analysis of variance (ANOVA) followed by Tukey’s comparison for (G) and (H). Please note that Tukey’s comparison gave *P* > 0.05 for the MRTX-isotype versus MRTX–anti-NK1.1. We performed a focused comparison between the MRTX-isotype and MRTX–anti-NK1.1 groups using an independent *t* test. This secondary analysis yielded a statistically significant result *P* = 0.039. Survival comparisons were made using Kaplan-Meier curves and the Mantel-Cox log-rank test for (I). *P < 0.05, **P < 0.01, and ***P < 0.001. ns, not significant.

To examine how KRAS^G12D^ inhibition alters the tumor immune microenvironment (TIME), we analyzed immune populations in orthotopic KPC (*Kras*^*G12D*/*+*^; *p53*^*R172H*/*+*^; *Pdx1-Cre^tg/+^*) organoids implanted in C57BL/6J mice treated with vehicle or the KRAS^G12D^ inhibitor MRTX1133. MRTX1133 markedly suppressed ERK signaling and tumor growth (fig. S1, A to C). Immune profiling revealed that MRTX1133 treatment led to a marked reduction in the accumulation of Gr-1^+^ MDSCs. Conversely, there was a documented trend toward enhanced infiltration of cytotoxic effectors, including NK cells and CD8^+^ T cells (Fig. 1, A and B, and fig. S1D).

To corroborate these results, we analyzed single-cell RNA sequencing (scRNA-seq) data from vehicle- and MRTX1133-treated murine KPC tumors [Gene Expression Omnibus (GEO): GSE228502]. NK cells from MRTX1133-treated tumors showed significantly higher expression of effector and activation markers, including *Gzmb*, *Prf1*, and *Klrk1* (fig. S1, E and F). In parallel, inhibitory receptor *Klrc1* was substantially down-regulated in NK cells from MRTX1133-treated tumors (fig. S1F). Flow cytometry analysis of orthotopic KPC tumors confirmed increased expression of Granzyme B (GZMB) and natural killer group 2 member D (NKG2D) and decreased expression of NKG2A in NK cells following MRTX1133 treatment (Fig. 1C). We also noted increased interferon-γ (IFN-γ) and Fas ligand (FASL) in CD8^+^ T cells from MRTX1133-treated tumors (Fig. 1C and fig. S1F), which is consistent with a previous report (*9*).

To test the functional importance of NK cells in the antitumor response to KRAS^G12D^ inhibition, NK cells were depleted using anti-NK1.1 antibody in vehicle- and MRTX1133-treated mice (Fig. 1D). Although NK cell depletion did not significantly affect MRTX1133-caused ERK signaling inhibition and cell apoptosis (indicated by phosphorylated ERK and cleaved caspase-3 staining; Fig. 1, E to G), NK cell depletion partially reversed the antitumor efficacy of MRTX1133 and significantly reduces survival compared to the MRTX1133-treated group in orthotopic KPC mice (Fig. 1, H and I). Notably, although NK cell depletion reduced the efficacy of MRTX1133, the survival rate remained significantly higher than the vehicle group (Fig. 1I). This indicates that while NK cells contribute to the drug’s effectiveness, they are not the sole drivers of the therapeutic response. The remaining protection likely stems from a combination of tumor-intrinsic effects and the recruitment of other immune cell populations. Together, these data indicate that KRAS^G12D^ inhibition restrains progression of KRAS^G12D^ PDAC in part by reprogramming the TIME, enhancing NK and CD8^+^ T cell activation while reducing MDSC accumulation.

### KRAS^G12D^ inhibition combined with NK cell therapy produces a synergistic antitumor response

**Fig. 2.**
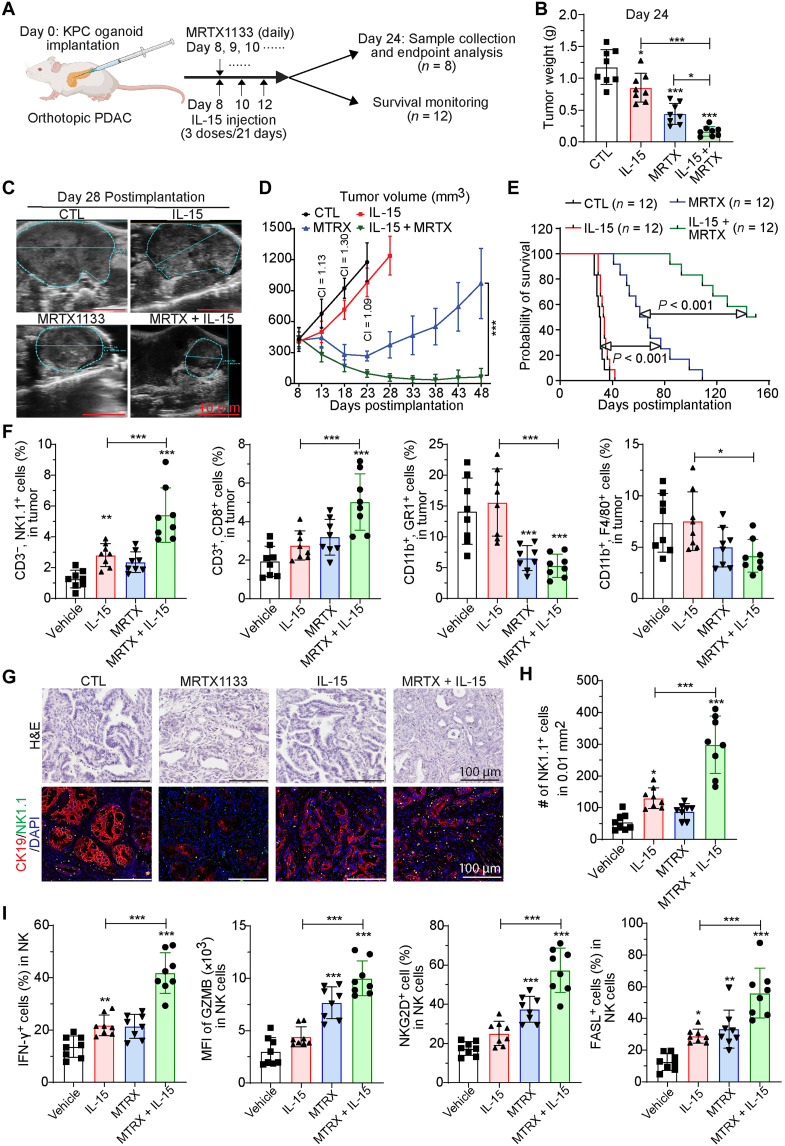
KRAS^G12D^ inhibitor in combination with NK cell therapy exhibits potent antitumor effect. (**A**) Schematic of the experimental design for evaluating combination therapy with the KRAS^G12D^ inhibitor MRTX1133 and NK cell activation cytokine IL-15 in orthotopic PDAC KPC tumors. (**B**) Statistical analysis of tumor weight from KPC tumors treated with vehicle, IL-15, MRTX1133, or MRTX1133 + IL-15 for 2 weeks. (**C**) Representative ultrasound images showing the largest cross sections of orthotopic KPC tumors from each treatment group. (**D** and **E**) Tumor growth curves (D) and Kaplan-Meier survival curves (E) of orthotopic KPC tumor–bearing mice. CI: bliss synergy score: >1, synergy; <1, antagonism; = 1, additive. (**F**) Flow cytometry analysis of the percentages of NK cells, CD8 T cells, MDSCs, and macrophages in orthotopic KPC tumors from indicated groups. (**G** and **H**) Representative hematoxylin and eosin (H&E) and IF staining for NK1.1 and corresponding statistical analyses in orthotopic KPC tumors. (**I**) Flow cytometry analysis of IFN-γ, GZMB, NKG2D, and FASL expression in tumor-infiltrating NK cells from the indicated treatment groups. Data are expressed as mean ± SEM. Statistical significance was determined using a two-tailed Student’s *t* test for (D) on day 48. Survival comparisons were made using Kaplan-Meier curves and the Mantel-Cox log-rank test for (E). One-way ANOVA followed by Tukey’s comparison for (F), (H), and (I). *P < 0.05, **P < 0.01, and ***P < 0.001. DAPI, 4′,6-diamidino-2-phenylindole.

The above results indicate that KRAS^G12D^ blockade appears to remodel the TIME into one that is permissive for NK cell activity. To test whether this remodeling enhances the efficacy of NK-directed therapy, we evaluated the combination of the MRTX1133 with NK-stimulating cytokine IL-15 in KRAS^G12D^-mutant PDAC and CRC models (Fig. 2A and fig. S2A). Across both tumor types, IL-15 alone showed a limited antitumor effect, while IL-15 markedly potentiated MRTX1133’s antitumor efficacy compared with MRTX1133 alone (Fig. 2, B to D, and fig. S2, B to E). Combination treatment also significantly extended survival of PDAC-bearing mice relative to single-agent cohorts (Fig. 2E).

Immunoprofiling revealed that MRTX1133 amplified the IL-15–driven expansion and concomitantly reducing accumulation of CD11b^+^, Gr-1^+^ myeloid cells (Fig. 2, F to H, and fig. S2, F and G). MRTX1133 treatment also enhanced the IL-15–induced activation of NK cells (Fig. 2I and fig. S2H). In addition, the combined regimen also increased CD8^+^ T cell infiltration and decreased macrophage presence in PDAC and CRC tumors (Fig. 2F and fig. S2F).

Since IL-15 activates both NK and CD8^+^ T cells, and MRTX1133 also further boosts Fas-positive CD8^+^ T cell infiltration (Fig. 1C and fig. S1D), we used selective depletion to isolate the cellular basis of their combined efficacy (fig. S3, A and B). Although CD8^+^ T cell depletion partially attenuated the antitumor response, the effect was not statistically significant. In contrast, NK cell depletion caused a substantial loss of therapeutic activity (fig. S3, C to F). These results indicate NK cells, rather than CD8^+^ T cells, as the primary drivers of synergy between MRTX1133 and IL-15. Ultimately, this indicates that KRAS^G12D^ inhibition fosters an NK-permissive microenvironment, suggesting that combining this inhibition with NK-directed therapies offers enhanced control of KRAS^G12D^-mutant tumors.

### KRAS^G12D^ inhibition increases the sensitivity of tumor cells to NK cell cytotoxicity

**Fig. 3.**
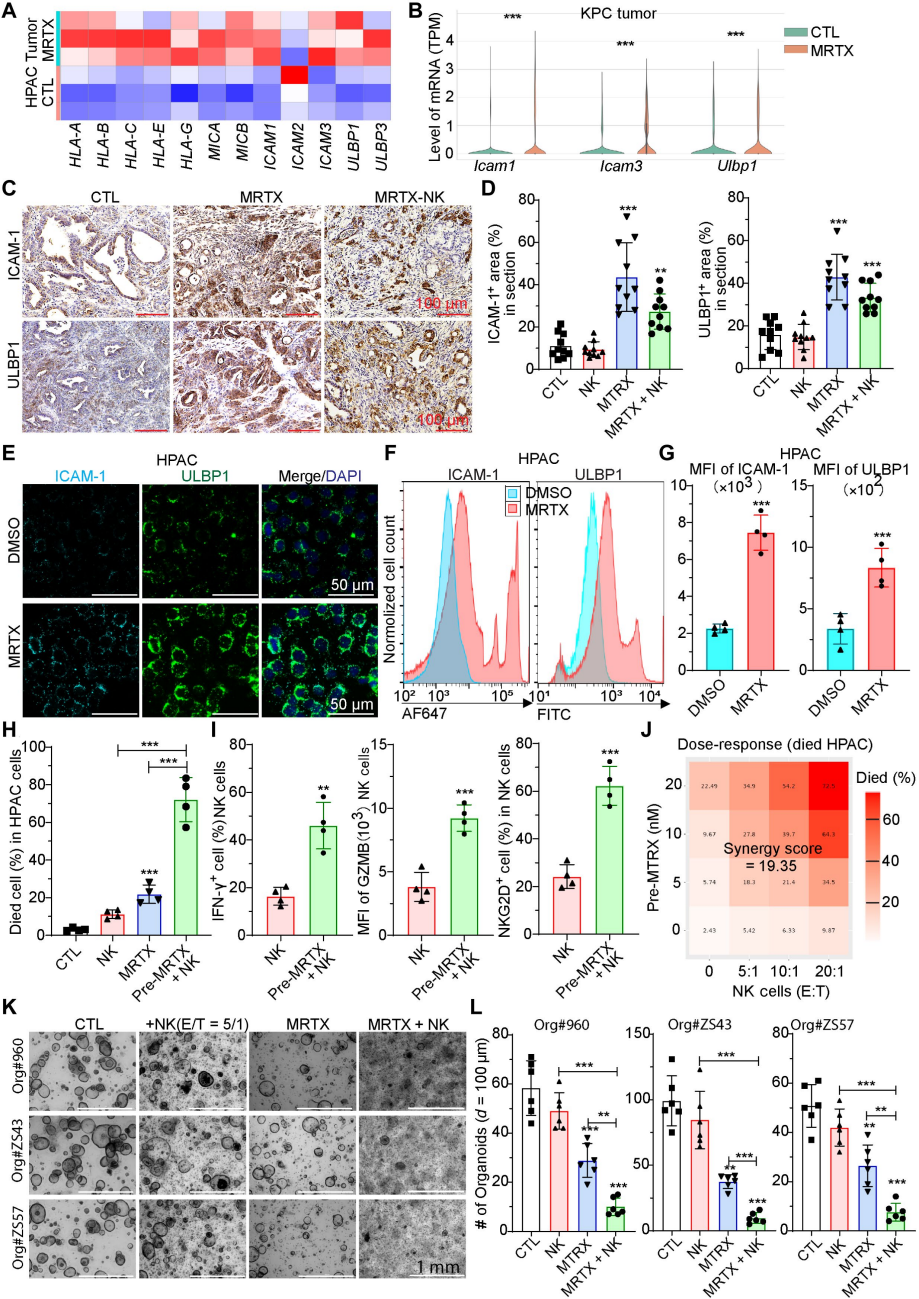
KRAS^G12D^ inhibition increases the sensitivity of tumor cell to NK cell cytotoxicity. (**A**) Heatmap analysis of key ligands associated with NK cell–mediated cytotoxicity in HPAC xenograft tumors following MRTX1133 treatment (GEO: GSE201412). (**B**) Violin plots showing the expression of *Icam1*, *Icam3*, and *Ulbp1* in KPC tumor cells treated with vehicle or MRTX1133 (scRNA-seq data from GEO: GSE228502). (**C** and **D**) Representative IHC staining and quantification of ICAM-1 and ULBP1 in orthotopic PDX tumors treated with vehicle, MRTX1133, or MRTX1133 + NK cell infusion. (**E** to **G**) Representative IF staining (E), flow cytometry plots (F), and quantification (G) of ICAM-1 and ULBP1 in HPAC cells treated with vehicle or MRTX1133 (20 nM, 48 hours). (**H**) Cytotoxicity assay showing the percentage of dying HPAC cells after pretreatment with MRTX1133 for 24 hours followed by coculture with NK cells for 20 hours. (**I**) Expression of IFN-γ, GZMB, and NKG2D in NK cells after coculture with vehicle- or MRTX1133-pretreated HPAC cells. (**J**) Synergy analysis of MRTX1133 and NK cell–mediated cytotoxicity on HPAC cells. E:T, effector cells:tumor cells. (**K** and **L**) Representative images (K) and quantification (L) of KRAS^G12D^-mutated PDAC organoids cocultured with or without NK cells (E:T ratio = 20:1) and treated with vehicle or MRTX1133 (20 nM, 7 days). Wilcoxon rank-sum tests were used for (B). Data are expressed as mean ± SEM. Statistical significance was determined using one-way ANOVA followed by Tukey’s comparison for (D), (H), and (L) and two-tailed Student’s *t* test for (G) and (I). **P < 0.01 and ***P < 0.001. TPM, transcripts per million. FITC, fluorescein isothiocyanate. AF647, Alexa Fluor 647.

To elucidate the molecular mechanisms underlying the synergistic antitumor effect of KRAS^G12D^ inhibition and adoptive NK cell therapy, we compared the mRNA levels of major NK cell activation and inhibitory ligands in HPAC cells-derived xenograft tumors following vehicle or MRTX1133 treatment (GEO: GSE201412). MRTX1133 treatment led to an up-regulation of several NK cell activation ligands (*MICA/B*, *ICAMs*, and *ULBP*s), alongside inhibitory ligands [major histocompatibility complex I (MHC-I) molecules] (Fig. 3A). The scRNA-seq data from KPC tumors (GEO: GSE228502) and PDAC organoid–derived xenograft tumors confirmed the increased expression of *ICAMs* and *ULBP1* in cancer cells (Fig. 3B and fig. S4, A and B). Further validation through immunohistochemical staining demonstrated a significant increase in ICAM1 and ULBP1 expression levels in MRTX1133-treated PDAC organoid–derived xenograft tumors and syngeneic KPC PDAC mouse tumors (Fig. 3, C and D, and fig. S4C). In addition, immunofluorescence (IF) staining and flow cytometry analysis in KRAS^G12D^-mutant HPAC and PANC1 cells indicated that MRTX1133 treatment substantially increased the expression of ICAM1 and ULBP1 (Fig. 3, E to G, and fig. S4, D and E). These findings collectively suggest that KRAS^G12D^ inhibition may directly enhance the sensitivity of cancer cells to NK cells through the up-regulation of NK cell–activating ligands, such as ICAMs and ULBPs.

To further investigate the direct effect of KRAS^G12D^ inhibition on the sensitivity of cancer cells to NK cells, KRAS^G12D^-mutant HPAC and PANC1 cells were pretreated with MRTX1133 and subsequently cocultured with NK cells derived from peripheral blood mononuclear cells (PBMCs) of healthy donors. The results indicated that MRTX1133 pretreatment significantly sensitized HPAC and PANC1 cells to NK cell–mediated cytotoxicity (Fig. 3H and fig. S4F). This sensitization was accompanied by a notable elevation of IFN-γ, GZMB, and NKG2D expression in NK cells that were stimulated by the MRTX1133-pretreated cancer cells (Fig. 3I and fig. S4G). Furthermore, MRTX1133 pretreatment demonstrated a strong synergistic effect with NK cell–mediated cytotoxicity on both HPAC and PANC1 cells (Fig. 3J and fig. S4H). Similar results were observed in KRAS^G12D^-mutant PDAC organoids treated with MRTX1133 and cocultured with NK cells from PBMCs of healthy donors (Fig. 3, K and L), reinforcing the direct impact of KRAS^G12D^ inhibition on enhancing the susceptibility of cancer cells to NK cell activity.

### KRAS^G12D^ inhibition promotes IFN-γ response via impairing IFNGR lysosome degradation

**Fig. 4.**
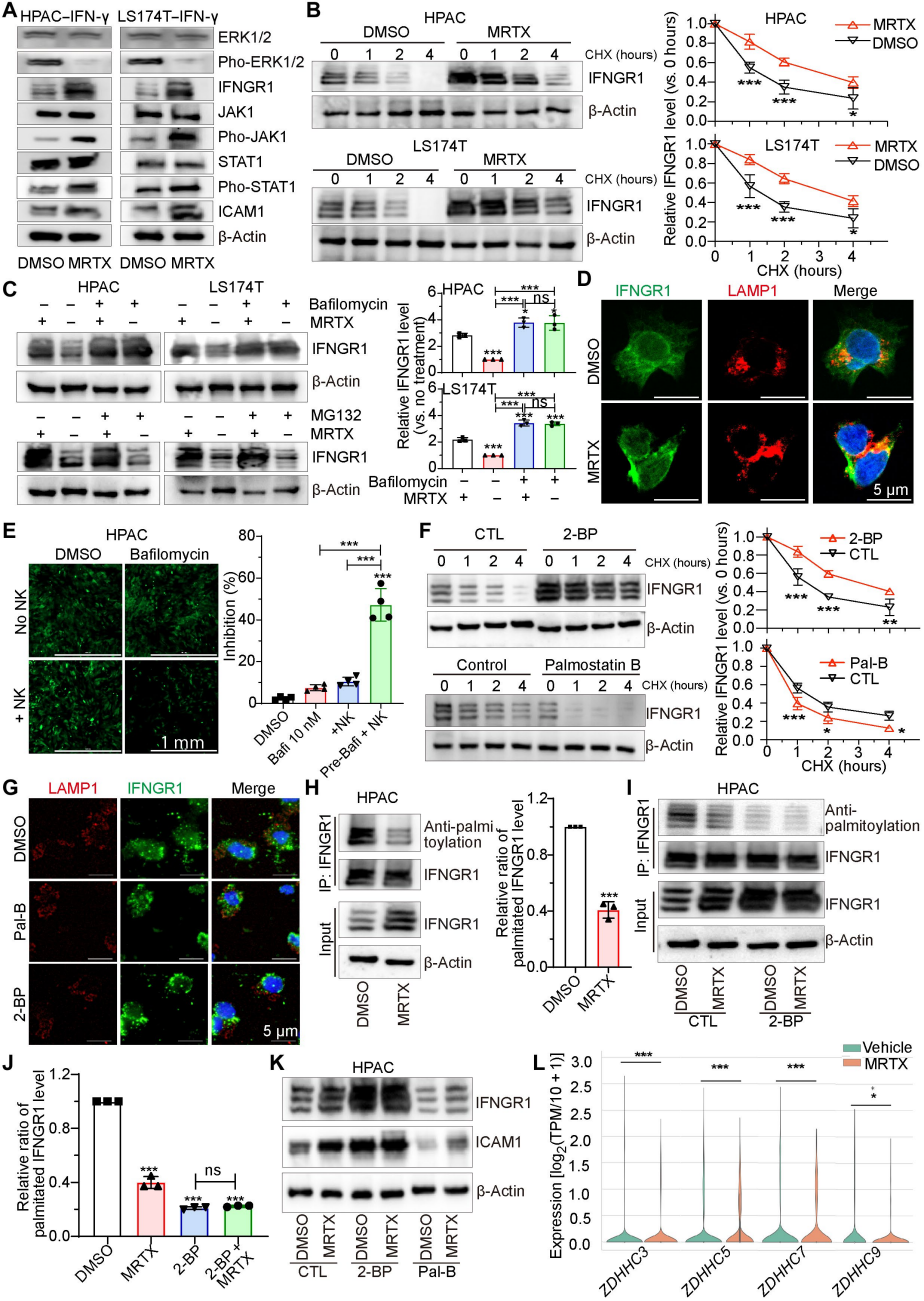
KRAS^G12D^ inhibition enhances IFNGR1 stability by impairing its palmitoylation. (**A**) Immunoblot analysis of ERK1/2, p-ERK1/2, IFNGR1, JAK1, p-JAK1, STAT1, p-STAT1, ICAM1, and β-actin in HPAC (PDAC) and LS174T (CRC) cells after treatment with MRTX1133 for 48 hours. (**B** and **C**) Immunoblot and quantification of IFNGR1 expression following the indicated treatments. (**D**) Representative IF images showing the effect of MRTX1133 on the colocalization of IFNGR1 (red) with the lysosomal marker LAMP1 (green) in LS174T cells. Nuclei were stained with DAPI (blue). (**E**) Representative IF images and quantitative analysis showing the effect of the lysosomal inhibitor bafilomycin A1 (Bafi, 10 nM) on the sensitivity of HPAC cells to NK cell–mediated killing. HPAC cells were preincubated with calcein AM, treated with Bafi for 24 hours, and then cocultured with NK cells (target: NK = 1:10) for 20 hours. (**F**) Immunoblot analysis and quantification of IFNGR1 expression following treatment with palmitoylation inhibitors 2-bromopalmitate (2-BP) and palmostatin B (Pal-B). (**G**) Representative IF images showing the effect of 2-BP and Pal-B on IFNGR1 lysosomal colocalization in HPAC cells. (**H**) Immunoblot analysis and quantification of total IFNGR1 (input) and palmitoylated IFNGR1 [co-immunoprecipitation (co-IP), relative palmitoylated-IFNGR1 ratio = anti-palmitoylation/anti-IFNGR1 in IP part] in HPAC cells after treatment with MRTX1133 for 48 hours. (**I** and **J**) Immunoblot analysis and quantification of total IFNGR1 (input) and palmitoylated IFNGR1 (co-IP) in HPAC after treatment with 2-BP and MRTX1133 for 48 hours. (**K**) Immunoblot analysis of IFNGR1 and ICAM1 in HPAC cells after treatment with 2-BP, Pal-B, and MRTX1133. (**L**) Violin plots showing the expression of palmitoyl transferases in *EPCAM*^+^ PDAC cells from vehicle- or MRTX1133-treated PDX tumors. Data are expressed as mean ± SEM. Statistical significance was determined using two-tailed Student’s *t* test for (B), (F), and (H). One-way ANOVA followed by Tukey’s comparison for (C), (E), and (J). Wilcoxon rank-sum tests were used for (L). *P < 0.05, **P < 0.01, and ***P < 0.001. ns, not significant. CHX, cycloheximide.

To investigate the mechanism by which KRAS^G12D^ inhibition enhances NK cell activation ligands, such as ICAM1, we analyzed single-cell transcriptome data from cancer cells within PDAC organoid–derived xenograft tumors after MRTX1133 treatment. MRTX1133 administration resulted in a significant up-regulation of *ICAM1* and a notable enrichment of IFN-γ response signaling, a primary upstream regulator of ICAMs and ULBPs, in cancer cells (fig. S5, A to C). While MRTX1133 did not elevate the mRNA levels of *IFNGR1* or *IFNGR2* (fig. S5, C and D), it did increase IFN-γ receptor 1 (IFNGR1) protein expression and augmented IFN-γ–induced Janus kinase 1 (JAK1)–signal transducer and activator of transcription 1 (STAT1) signaling activation and subsequent ICAM1 expression (Fig. 4A and fig. S5E). Further evidence supporting the role of the IFNGR-JAK-STAT pathway in MRTX1133-induced increase of ICAM1 was obtained using JAK inhibitors ruxolitinib and baricitinib, which successfully abrogated the MRTX1133-induced increase of ICAM1 (fig. S5F), thereby confirming that MRTX1133 up-regulates ICAM1 expression via the IFNGR-JAK-STAT signaling cascade.

The disparity between increased IFNGR1 protein levels and unchanged mRNA levels following MRTX1133 treatment may imply a regulatory effect on IFNGR1 protein stability rather than transcriptional activity. To validate this, we quantified the half-life of IFNGR1 in human KRAS^G12D^-mutated HPAC and LS174T cells. Inhibition of de novo protein synthesis with cycloheximide revealed that the IFNGR1 half-life in dimethyl sulfoxide (DMSO)–treated control cells was fourfold shorter than in MRTX1133 pretreated cells (Fig. 4B), suggesting that MRTX1133 may enhance IFNGR1 protein stability and hinder its degradation.

To elucidate the predominant protein degradation pathway, we challenged DMSO and MRTX1133 pretreated HPAC (a PDAC cell line) and LS174T (a CRC cell line) cells with bafilomycin (a lysosome inhibitor) and MG132 (a proteasome inhibitor). Bafilomycin successfully rescued IFNGR1 protein expression in DMSO-treated controls, while MG132 failed (Fig. 4C), indicating that IFNGR1 protein is primarily transported to and degraded within lysosomes. Subsequent IF staining for IFNGR1 and LAMP1 (a lysosome marker) in DMSO- and MRTX1133-treated cells further supported this by showing that MRTX1133 treatment reduced IFNGR1 localization in lysosomes, as evidenced by decreased costaining of IFNGR1 and LAMP1, even MRTX1133 treatment markedly increased lysosomal quantity (Fig. 4D and fig. S5, G and H). Inhibition of lysosome activity with bafilomycin increases the sensitivity of tumor cells to NK cell cytotoxicity (Fig. 4E and fig. S5I). Collectively, these findings provide compelling evidence that KRAS^G12D^ inhibition impairs lysosome-mediated IFNGR1 degradation, thereby amplifying the IFN-γ response signal.

### KRAS^G12D^ inhibition enhances IFNGR1 stability by attenuating its palmitoylation

Recent study has established a link between the palmitoylation of IFNGR1 and its subsequent stability and degradation within the lysosome (*21*). Building on this foundation, we sought to elucidate the connection between IFNGR1 palmitoylation and its degradation following KRAS^G12D^ inhibition. Our data confirmed that IFNGR1 palmitoylation is a critical determinant of its cellular fate in KRAS^G12D^-mutant cells. Treatment of HPAC cells with 2-bromopalmitate (2-BP, an inhibitor of palmitoylation) resulted in a marked reduction of IFNGR1 localization to lysosomes and a corresponding increase in its overall stability (Fig. 4, F and G). Conversely, treatment of palmostatin B (Pal-B, an inhibitor of depalmitoylation) led to increased lysosomal targeting and accelerated degradation of IFNGR1 (Fig. 4, F and G). These results collectively validate that IFNGR1 palmitoylation serves as a signal for its lysosomal sorting and subsequent degradation.

Crucially, treatment with MRTX1133 significantly increased total IFNGR1 protein levels while concurrently decreasing the amount of palmitoylated IFNGR1 in both HPAC and LS174T cells (Fig. 4H and fig. S6A). When cells were cotreated with the palmitoylation inhibitor 2-BP, administration of MRTX1133 did not produce any further increase in total IFNGR1 or reduction in its palmitoylated form (Fig. 4, I and J, and fig. S6, B and C). This lack of an additive effect suggests that MRTX1133 and 2-BP likely share a common mechanism of action, specifically the inhibition of palmitoylation, to achieve IFNGR1 stabilization. To further substantiate that MRTX1133 may directly impede the palmitoylation reaction rather than promoting depalmitoylation, we co-used MRTX1133 with the depalmitoylation inhibitor Pal-B. As expected, Pal-B treatment increased IFNGR1 palmitoylation levels (fig. S6, B and C). However, MRTX1133 still effectively reduced IFNGR1 palmitoylation and increased the IFNGR1 level when depalmitoylation was inhibited (fig. S6, B and C). These results provide compelling evidence that KRAS^G12D^ inhibition–caused attenuation of IFNGR1 palmitoylation primarily functions by attenuating the initial palmitoylation reaction. Consistent with this, while Pal-B treatment reduced the overall expression of IFNGR1 and ICAM1, subsequent KRAS^G12D^ inhibition with MRTX1133 was still capable of elevating IFNGR1 levels and partially restoring the IFN-γ–induced increase of ICAM1 (Fig. 4K).

We then explored how KRAS^G12D^ inhibition might regulate the cellular palmitoylation machinery. The palmitoylation process is governed by two principal factors: the availability of the palmitate–coenzyme A substrate and the expression and activity of palmitoyltransferases (enzymes that catalyze the palmitoylation reaction). Analysis of the single-cell transcriptomics in normal pancreatic acinar and ductal cells and malignant cells [Genome Sequence Archive (GSA): CRA001160] revealed a significant overexpression of four specific palmitoyltransferases, *ZDHHC3*, *ZDHHC5*, *ZDHHC7*, and *ZDHHC9*, in the PDAC cells comparing to acinar and ductal cells (fig. S6, D and E). In our patient-derived xenograft (PDX) model of PDAC, treatment with MRTX1133 markedly reduced the expression of these four enzymes in tumor cells (Fig. 4L and fig. S6F). The quantitative polymerase chain reaction analysis confirmed that MRTX treatment significantly down-regulated the mRNA expression of *ZDHHC3*, *ZDHHC5*, *ZDHHC7*, and *ZDHHC9* in both in vitro cell cultures and in vivo tumor models (fig. S7, A to C). In parallel, we investigated the impact of KRAS^G12D^ inhibition on the palmitoylation substrate pool. Our recent studies have demonstrated that MRTX1133 treatment shifts cellular metabolism away from glucose utilization and toward fatty acid oxidation. Using ^13^C-labeled metabolic tracing, we confirmed that MRTX1133 inhibited the de novo synthesis of palmitic acid from glucose (fig. S7, D and E). Furthermore, metabolic flux tracing of ^13^C-labeled palmitate showed that MRTX1133 accelerates its oxidative catabolism (fig. S7F). This dual effect, decreased synthesis and increased degradation, may result in a diminished intracellular pool of palmitic acid available for the palmitoylation reaction. In summary, these findings indicate that the inhibition of KRAS^G12D^ enhances IFNGR1 stability may be through a multifaceted mechanism that related to suppression of IFNGR1 palmitoylation by concurrently reducing the expression of palmitoyltransferases and depleting the cellular supply of the essential palmitate substrate.

### KRAS^G12D^ inhibition impairs tumor-induced local and systemically immunosuppressive effect

As KRAS^G12D^ inhibition also diminished myeloid population accumulation and increased infiltration and activation of NK and CD8^+^ T cells (Fig. 1A and fig. S1D), the underlying mechanism was then investigated. Cytokine array results indicated that monocyte chemotactic protein 1 (MCP-1), IL-2, GM-CSF (CSF2), IL-7, IL-6, and IL-1a levels were significantly decreased in tumor interstitial fluid (TIF) of orthotopic tumor treated by MRTX1133 (fig. S8A). Analysis of scRNA-seq data (GSA: CRA001160) from human normal pancreases and pancreatic tumors revealed that *CSF2* (coding GM-CSF) was mainly expressed by pancreatic cancer cells among above six significantly altered cytokines (fig. S8, B to D). Moreover, the expression of *CSF2* decreased in mRNA, conditioned medium, and cell lysis from MRTX1133-treated KPC and HPAC cells (fig. S8E). This result was consistent with previous findings that oncogenic *Kras* mutation induced GM-CSF production to recruit suppressive myeloid cells and promote cancer progression (*8*, *22*, *23*). In addition, the receptor of GM-CSF (*CSF2RA*) was specifically expressed by myeloid cells in pancreatic tumors (fig. S8F). Together, these results indicate that KRAS^G12D^ inhibition down-regulates CSF2 in cancer cells to reduce MDSC accumulation, thereby promoting infiltration and activation of NK cell and CD8^+^ T cell.

Beyond alterations within the TIF (fig. S8A), cytokine array analyses of plasma samples revealed a notable reduction in the concentrations of critical immunoregulatory cytokines, including IL-6, GM-CSF (CSF2), IL-2, granulocyte colony-stimulating factor, and macrophage inflammatory protein-1/2 (MIP-1/2), in orthotopic KPC tumors treated with MRTX1133 (fig. S9A), suggesting that KRAS^G12D^ inhibition may also favorably modulate the tumor-induced systemic immune response. Consistent with recent findings, KPC tumors induced systemic immune reaction in tumor-bearing hosts, indicating by splenomegaly (a common feature in patients with advanced PDAC), expansion of myeloid populations (macrophages and MDSCs), and reduction of lymphocyte subsets (T cells, B cells, and NK cells) in both the peripheral blood and splenic compartments when compared to healthy control mice (fig. S9, B to E). Crucially, the administration of MRTX1133 effectively reversed these tumor-induced systemic immunosuppressive phenotypes, leading to a remarkable restoration of immune cell homeostasis, promoting a significant increase in the ratios of lymphocyte subsets and decrease in the ratios of myeloid populations within both the circulatory system and the spleens. These restored immune cell ratios closely approximated those observed in healthy mice (fig. S9, B to E), further underscoring the systemic immunomodulatory efficacy of KRAS^G12D^ inhibition.

### KRAS^G12D^ inhibition restores systemic NK cell abundance and function

**Fig. 5.**
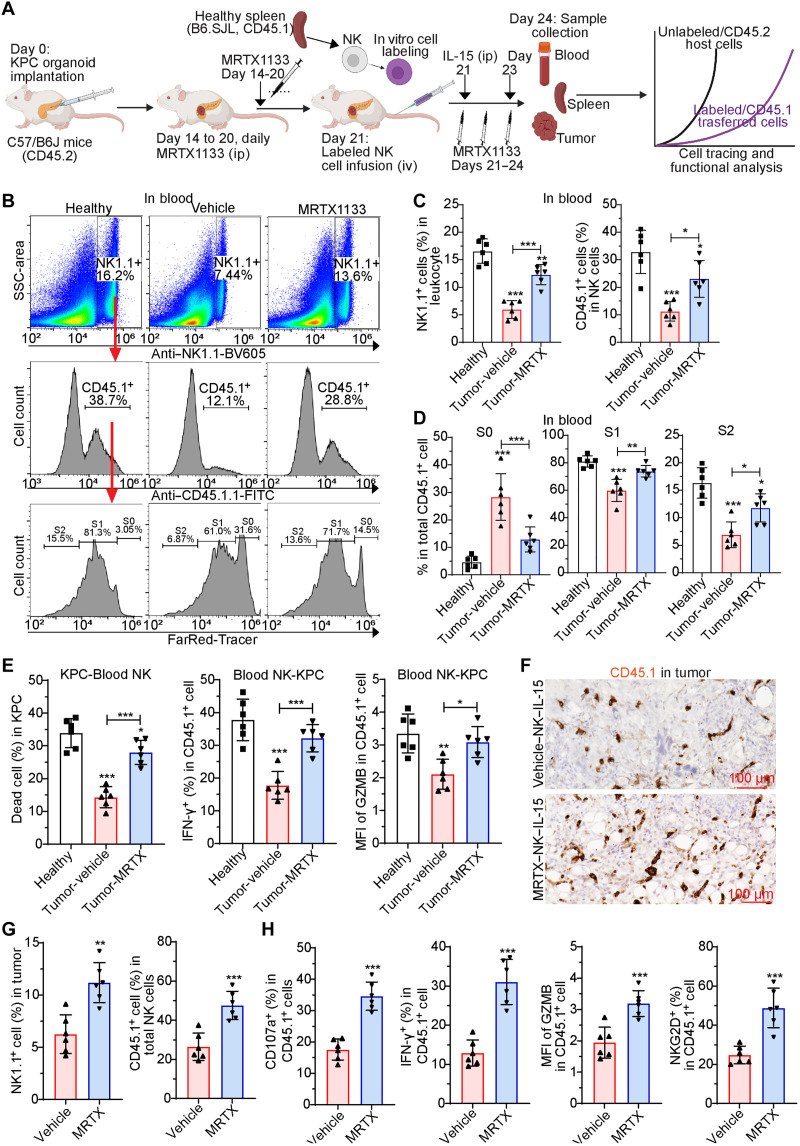
KRAS^G12D^ inhibition reverses tumor-induced systemic suppression of endogenous and adoptively transferred NK cells. (**A**) Schematic of the experimental design for tracing adoptively transferred NK cells and determining the effect of MRTX1133 treatment in the orthotopic KPC PDAC model. (**B** and **C**) Representative flow cytometry plots (B) and quantification (C) of total (NK1.1^+^) and adoptively transferred (NK1.1^+^, CD45.1^+^) NK cells in the blood of healthy and tumor-bearing mice. (**D**) Quantification of FarRed Tracer signal intensity, indicating the proliferative status of adoptively transferred NK cells in blood. (**E**) Ex vivo cytotoxicity assay showing the percentage of dying KPC cells following coculture with adoptively transferred NK cells isolated from blood. Expression of IFN-γ and GZMB in these NK cells was also analyzed. (**F**) Representative IHC staining of CD45.1 in tumor tissues from KPC tumor–bearing mice that received adoptively transferred NK cells (CD45.1^+^) and were treated with IL-15 in combination with vehicle or MRTX1133. (**G**) Quantification of total (NK1.1^+^) and adoptively transferred (CD45.1^+^) NK cells in KPC tumors from the indicated treatment groups. (**H**) Expression of CD107a, IFN-γ, GZMB, and NKG2D in adoptively transferred NK cells from KPC tumors of the indicated treatment groups. Data are expressed as mean ± SEM. Statistical significance was determined using one-way ANOVA followed by Tukey’s comparison for (C) to (E) and two-tailed Student’s *t* test for (G). *P < 0.05, **P < 0.01, and ***P < 0.001. ip, intraperitoneal; iv, intravenous.

To further investigate the systemic effects of KRAS^G12D^ inhibition on NK cells, NK cells from healthy B6-CD45.1 mice were adoptively transferred into KPC tumor–bearing C57BL/6J (CD45.2) mice, which were pretreated with or without MRTX1133 and subsequently treated with MRTX1133 and IL-15 (Fig. 5A). A significant reduction in the fractions of both total (NK1.1^+^) and adoptively transferred (CD45.1^+^) NK cells was observed in the circulatory system and spleens of vehicle-treated tumor-bearing mice when compared to healthy control mice (Fig. 5, B and C, and fig. S9F). Furthermore, using cell tracer dividing assays, it was determined that adoptively transferred NK cells exhibited suppressed proliferative capacity within KPC tumor–bearing mice, indicating a tumor-induced systemically inhibitory effect on NK cells (Fig. 5, B and D). However, the administration of MRTX1133 elicited a marked reversal of these suppressive trends. Treatment with MRTX1133 significantly increased the abundance of both total and adoptively transferred NK cells in the blood and spleens of tumor-bearing mice when contrasted with vehicle-treated controls (Fig. 5, B and C, and fig. S9F). Concurrently, the proliferation of adoptively transferred NK cells was notably accelerated in MRTX1133-treated tumor-bearing mice compared to their vehicle-treated counterparts (Fig. 5, B and D). Beyond mere quantitative changes, adoptively transferred NK cells, isolated from the blood and spleens of MRTX1133-treated tumor-bearing mice, demonstrated significantly heightened cytotoxicity against KPC cells in vitro, with increased expression of IFN-γ and GZMB relative to NK cells from vehicle-treated tumor-bearing mice, this enhanced cytotoxicity was found to be comparable to that observed in adoptively transferred NK cells obtained from the blood of healthy mice (Fig. 5E and fig. S9G), suggesting a robust restoration of NK cell effector function.

Crucially, MRTX1133 and IL-15 treatment markedly elevated the infiltration of both total and adoptively transferred NK cells into orthotopic KPC tumors (Fig. 5, F and G). The tumor-infiltrating CD45.1^+^ NK cells also showed significantly increased expression of activation markers (CD107a, IFN-γ, GZMB, and NKG2D) (Fig. 5H). These results provide compelling evidence that KRAS^G12D^ inhibition effectively counteracts the local and systemic immunosuppressive environment induced by the tumor and underscores the significant potential of KRAS^G12D^ inhibition to strengthen antitumor immunity by rejuvenating NK cell–mediated responses.

### KRAS^G12D^ inhibition improves the antitumor efficacy of adoptive NK cell therapy

**Fig. 6.**
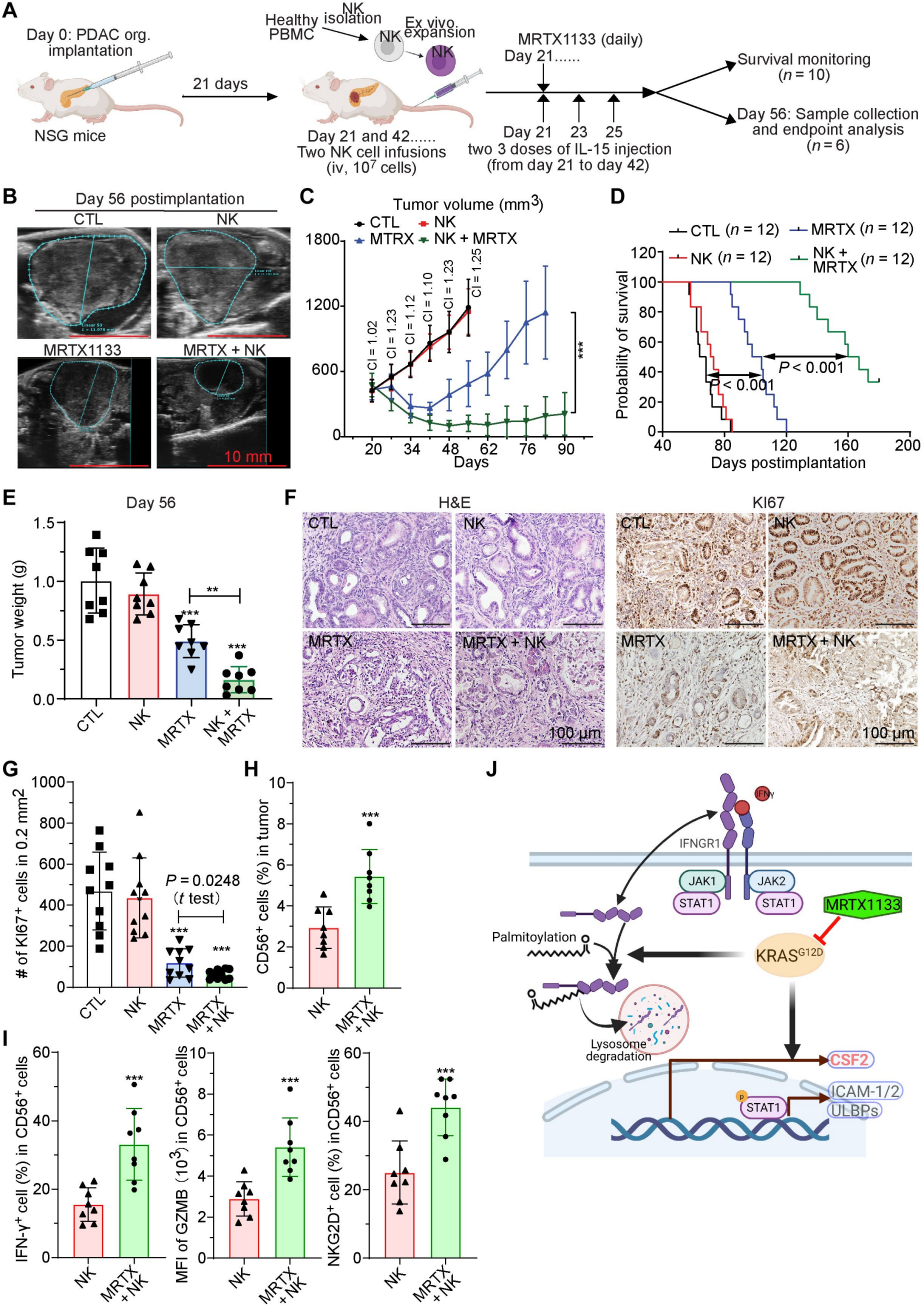
KRAS^G12D^ inhibitor improves the antitumor efficacy of NK cell therapy. (**A**) Schematic of the experimental design for evaluating adoptive NK cell transfer in combination with MRTX1133 in an orthotopic pancreatic PDX tumor model. (**B**) Representative ultrasound images of orthotopic PDX tumors from the indicated treatment groups. (**C** to **E**) Analysis of tumor weight (C), tumor growth curves (D), and Kaplan-Meier survival curves (E) of orthotopic PDX tumor–bearing mice. Combination index (CI): bliss synergy score: >1, synergy; <1, antagonism; = 1, additive. (**F** and **G**) Representative H&E and Ki67 staining images and corresponding quantification of Ki67-positive cells in PDX tumor tissues. (**H**) Flow cytometry analysis showing the percentage of NK cells in orthotopic PDX tumors. (**I**) Expression of IFN-γ, GZMB, and NKG2D in tumor-infiltrating NK cells from the indicated treatment groups. (**J**) Graphical mechanistic model: Oncogenic KRAS^G12D^ abolishes antitumor immunity by increasing GM-CSF production, which drives immunosuppressive myeloid cell infiltration, and by accelerating IFNGR1 palmitoylation and degradation, which impairs IFN-γ response signaling. Inhibition of KRAS^G12D^ by MRTX1133 reduces GM-CSF production, remodeling the TME to one that is permissive for NK cell activity, and prevents IFNGR1 degradation by counteracting palmitoylation. This enhances the expression of NK cell–activating ligands, thereby increasing tumor cell sensitivity to NK cell–mediated killing and restoring effective antitumor immunity. (J) Created in BioRender. Tuo Group (2026) https://BioRender.com/oy1vgea. Data are expressed as mean ± SEM. Statistical significance was determined using two-tailed Student’s *t* test for (C) on day 83, (H), and (I). Survival comparisons were made using Kaplan-Meier curves and the Mantel-Cox log-rank test for (E). One-way ANOVA followed by Tukey’s comparison for (E) and (G). **P < 0.01 and ***P < 0.001.

Given the extensive clinical investigation into adoptive NK and chimeric antigen receptor natural killer (CAR-NK) cell therapies for patients with PDAC and patients with CRC (NCT06730009, NCT06503497, NCT04319757, NCT05955157, NCT05213195, NCT05400122, etc.), we sought to determine whether targeting the KRAS^G12D^ mutation could synergistically augment the efficacy of this immunotherapeutic approach. We established orthotopic PDX models by using KRAS^G12D^-mutant PDAC and CRC organoids in NOD-SCID gamma (NSG) mice. These mice received infusions of human NK cells that were isolated from healthy donors and expanded ex vivo (Fig. 6A and fig. S10A). Our results indicated that adoptive NK cell monotherapy was insufficient to control tumor progression, as it did not significantly inhibit tumor growth or extend overall survival in either model (Fig. 6, B to D, and fig. S10, B to E). In contrast, the concurrent administration of MRTX1133 with adoptive NK cell transfer resulted in a powerful antitumor response and led to a significant reduction in tumor burden in both the PDAC and CRC PDX models (Fig. 6, B to D, and fig. S10, B to E), an effect that was substantially greater than that observed with MRTX1133 treatment alone. The combination of MRTX1133 and NK cells markedly prolonged the survival of tumor-bearing mice (Fig. 6D). Further investigation revealed that the combination treatment markedly suppressed tumor cell proliferation, as indicated by a significantly lower Ki67 index in the treated tumors (Fig. 6, F and G, and fig. S10, F and G). Moreover, immunophenotyping of the TME demonstrated that MRTX1133 administration enhanced NK cell infiltration into tumor tissues (Fig. 6H and fig. S10H) and promoted their activation status (Fig. 6I and fig. S10, I to K). Collectively, these findings suggest that KRAS^G12D^ inhibition can improve the antitumor effect of adoptively transferred NK cell therapy in KRAS^G12D^-driven cancers.

## DISCUSSION

The development of targeted inhibitors against specific KRAS mutations has marked a significant turning point in the treatment of solid tumors, historically deemed “undruggable.” While inhibitors of KRAS^G12C^ have demonstrated clinical utility (*24*), the KRAS^G12D^ mutation, highly prevalent in PDAC and CRC, has remained a formidable challenge. The recent emergence of specific KRAS^G12D^ inhibitors, such as MRTX1133, offers a promosing therapeutic avenue (*4*). Our study comprehensively investigates the impact of KRAS^G12D^ inhibition on the TIME and provides a strong rationale for combining this targeted therapy with NK cell–based immunotherapies to achieve synergistic antitumor effects.

The oncogenic activation of *KRAS* has been demonstrated that serves as the primary architect of the profound suppressive TIME characteristic of KRAS-mutated tumors, particularly in CRC and PDAC with KRAS^G12D^ mutation (*23*, *25*–*27*). This orchestration is achieved through a multifaceted signaling network that actively recruits and polarizes MDSCs and M2-like tumor associated macrophage (TAM) (*28*, *29*). By leveraging nuclear factor κB pathways, mutant KRAS triggers the secretion of potent chemokines and cytokines, such as CXCL1, CXCL2, CXCL5, and GM-CSF, effectively creating a cellular shield that prevents effective immunosurveillance (*28*, *29*). Beyond mere recruitment, KRAS signaling actively impairs the visibility and vulnerability of malignant cells by promoting programmed death-ligand 1 (PD-L1) expression and down-regulating MHC-I molecules (*30*, *31*). Furthermore, it uses epigenetic silencing mechanisms, using DNA methyltransferase 1 (DNMT1) and enhancer of zeste homolog 2 (EZH2) to repress the FAS death receptor, thereby rendering tumor cells resistant to the proapoptotic signals typically delivered by cytotoxic T cells (*25*, *26*).

A central finding of our work is that MRTX1133 profoundly remodels the TIME, shifting it from an immunosuppressive state to one that is permissive for robust antitumor immunity. We observed a significant increase in the infiltration of cytotoxic effector lymphocytes, including NK cells and CD8^+^ T cells, coupled with a marked reduction in MDSCs (granulocytic MDSCs). This finding is consistent with emerging reports on KRAS inhibitors, which suggest that beyond their direct cell-intrinsic effects, a substantial component of their efficacy is mediated through immunomodulation (*9*, *18*, *20*). Our scRNA-seq and flow cytometry data revealed that NK cells within MRTX1133-treated tumors exhibited a highly activated phenotype, characterized by increased expression of effector molecules like GZMB and PRF1, and activation receptors such as NKG2D, alongside a down-regulation of the inhibitory receptor NKG2A. This enhanced activation state suggests that KRAS^G12D^ inhibition primes NK cells for more effective tumor cell recognition and elimination. Notably, although NK cell depletion reduced survival rates compared to the MRTX1133 monotherapy group, survival remained superior to that of the vehicle-treated control. This suggests that while NK cells are important effectors, other mechanisms, including tumor-intrinsic factors and the recruitment of additional immune cell subsets, contribute to the observed therapeutic benefits.

Our investigation into the mechanism underlying this immune reprogramming pointed to a KRAS^G12D^-dependent regulation of cytokine production by cancer cells. Specifically, we identified a significant reduction in GM-CSF (CSF2) secretion from tumor cells following MRTX1133 treatment. Oncogenic KRAS has been previously shown to drive GM-CSF production (*8*), which, in turn, promotes the recruitment and expansion of immunosuppressive myeloid populations like MDSCs (*32*, *33*). By down-regulating GM-CSF, KRAS^G12D^ inhibition effectively dismantles a key axis of myeloid-mediated immunosuppression, thereby alleviating the barrier to NK and T cell infiltration and function. This finding mechanistically links the direct targeting of the KRAS oncogenic driver with the favorable reshaping of the immune landscape. The functional significance of this enhanced NK cell activity was confirmed through depletion experiments. NK cell depletion partially reversed the overall antitumor efficacy of MRTX1133, demonstrating that ensuing NK cell–mediated cytotoxicity is a crucial component for achieving maximal and sustained tumor control. This partial dependency highlights the multifaceted nature of the response to KRAS^G12D^ inhibition and suggests that strategies aimed at further augmenting NK cell function could unlock even greater therapeutic potential.

Furthermore, our study reveals that the immunomodulatory effects of KRAS^G12D^ inhibition are not confined to the local tumor milieu but extend systemically. Tumor burden, including PDAC, is known to induce profound systemic immunosuppression, characterized by a peripheral expansion of myeloid cells at the expense of reduced lymphocytes (*34*). Systemic immunity has been shown to be required for effective cancer immunotherapy (*35*). MRTX1133 treatment was able to reverse these systemic aberrations, restoring immune cell homeostasis in the peripheral blood and spleen to levels comparable to those in healthy mice. This systemic rescue has profound implications for immunotherapy (*36*), as we demonstrated that it counteracts the tumor-induced suppression of adoptively transferred NK cells. In MRTX1133-treated hosts, these NK cells exhibited improved survival, proliferation, and effector function, underscoring the potential of KRAS^G12D^ inhibition to create a more favorable systemic environment for cellular therapies. Our combination experiments, pairing MRTX1133 with the NK-stimulating cytokine IL-15 or with adoptive NK cell therapy, produced robust, synergistic antitumor responses that were far superior to monotherapy, leading to significant tumor regression and prolonged survival. Our findings demonstrate that KRAS^G12D^ inhibition can effectively “prime” the tumor, enhancing both the infiltration and activation of adoptively transferred NK cells.

The present study reveals another mechanism by which MRTX1133 treatment up-regulates the expression of NK cell–activating ligands, such as ICAM1 and ULBP1, on the surface of cancer cells and thereby directly sensitizes tumor cells to NK cell–mediated killing. This represents a complementary mechanism for observed synergy: Not only is the TIME made more hospitable for NK cells, but the tumor cells themselves become better targets. Our study further demonstrated that KRAS^G12D^ inhibition enhances the stability of IFNGR1 protein by inhibiting its lysosome-mediated degradation, which, in turn, amplifies the cellular response to IFN-γ, a key cytokine in the TIME, leading to increased activation of JAK-STAT signaling and transcription of IFN-γ response genes, including ICAM1 (*37*, *38*). Our mechanistic investigation revealed that the effect on IFNGR1 stability is mediated through the regulation of protein palmitoylation. KRAS^G12D^ signaling appears to promote IFNGR1 palmitoylation, which serves as a signal for its lysosomal degradation (*21*, *39*). Inhibition of KRAS^G12D^ attenuates this process through a dual mechanism: It reduces the expression of key palmitoyltransferase enzymes (*ZDHHCs*) and simultaneously depletes the intracellular pool of the palmitate substrate by shifting cellular metabolism from glucose utilization to lipid oxidation. This intricate mechanism directly links oncogenic signaling and metabolic reprogramming to the regulation of immune receptor stability and cancer cell immunogenicity.

Our findings integrate tumor-intrinsic changes with extrinsic modifications to the immune milieu, proposing a comprehensive dual-action model for tumor clearance following KRAS^G12D^ inhibition combined with NK cell therapy. The reduction of MDSC, driven by a decrease in GM-CSF production after MRTX1133 treatment, may act as a parallel mechanism that removes significant extrinsic barriers to immune infiltration. This alleviation of local immunosuppression works in concert with the IFNGR1/ICAM1 axis to ensure that NK cells are not only recruited to the tumor site in greater numbers but are also functionally persistent. By simultaneously increasing the intrinsic susceptibility of the tumor and dismantling the protective immunosuppressive shield of the microenvironment, KRAS^G12D^ inhibition provides a robust framework for overcoming the recalcitrant nature of PDAC, offering a sophisticated paradigm for future combinatorial immunotherapeutic strategies (graphical model).

In conclusion, our study provides a comprehensive preclinical validation for combining KRAS^G12D^ inhibition with NK cell–based immunotherapy. We demonstrate that MRTX1133 exerts a powerful, multipronged effect on the immune system: It alleviates local and systemic immunosuppression by reducing MDSCs, enhances the activation and function of endogenous and adoptively transferred NK cells, and directly sensitizes tumor cells to NK-mediated cytotoxicity by up-regulating activating ligands via a mechanism involving IFNGR1 stabilization. These findings provide a compelling mechanistic rationale for the clinical evaluation of combination strategies pairing KRAS^G12D^ inhibitors with NK cell–engaging therapies, such as adoptive NK cell transfer, CAR-NK cells, or NK-stimulating cytokines, to improve outcomes for patients with KRAS^G12D^-mutant cancers.

## MATERIALS AND METHODS

### Study design

#### Research objectives

The primary objective of this research was to investigate the immunomodulatory effects of KRAS^G12D^ inhibition and to determine its potential for synergistic combination with NK cell–based immunotherapies. Our prespecified hypotheses were as follows: (i) that targeted inhibition of oncogenic KRAS^G12D^ signaling would reprogram the immunosuppressive TME to a state more permissive for innate and adaptive antitumor immunity; and (ii) that MRTX1133 would sensitize KRAS^G12D^-mutant cancer cells to NK cell–mediated cytotoxicity. We further hypothesized that combining KRAS^G12D^ inhibition with NK cell–stimulating cytokines or adoptive NK cell therapy would yield superior antitumor efficacy compared to monotherapy. Following initial data analysis which indicated a significant up-regulation of IFN-γ response signaling, we formed a subsequent hypothesis: That KRAS^G12D^ inhibition enhances the stability of the IFNGR1 on cancer cells. Mechanistic investigations were then designed to test the hypothesis that this stabilization is mediated through the attenuation of IFNGR1 palmitoylation and its subsequent lysosomal degradation.

#### Research subjects and units of investigation

This study used a multiplatform approach involving cell lines, organoids, and animal models.

1) Cell cultures: In vitro experiments were performed using established human KRAS^G12D^-mutant PDAC cell lines (HPAC and PANC1), a CRC cell line (LS174T), and a murine PDAC cell line (KPC) derived from a genetically engineered mouse model. Primary human NK cells were isolated from PBMCs of healthy adult donors.

2) Organoid models: Three-dimensional organoid cultures were established from murine PDAC tumors arising in a *Kras^G12D/+^; p53^R172H/+^; Pdx1-Cre^tg/+^* (KPC) mouse model, as well as from human PDAC and CRC patient tumor tissues to create patient-derived organoids (PDOs). These models were used for in vitro coculture assays and for establishing PDX models.

3) Animal models: All animal experiments were conducted using 7- to 8-week-old mice. Immunocompetent wild-type C57BL/6J mice were used for syngeneic orthotopic tumor models with murine KPC organoids. For adoptive transfer and cell tracing studies, B6 Ptprca (B6 CD45.1) congenic mice were used as NK cell donors. Immunodeficient NSG mice were used to establish orthotopic PDX models from human PDOs.

#### Experimental design

The overall study used a controlled laboratory experimental design, integrating in vitro, ex vivo, and in vivo preclinical models to investigate the dual mechanisms of KRAS^G12D^ inhibition.

#### In vivo treatment studies

1) Syngeneic models: C57BL/6J mice bearing orthotopic KPC tumors were treated with vehicle or MRTX1133 to assess effects on tumor growth, survival, and the TIME.

2) Combination therapy models: To evaluate synergy, mice with orthotopic KPC or CRC tumors were treated with one of four regimens: vehicle, MRTX1133 alone, IL-15 alone, or the combination of MRTX1133 and IL-15. Similarly, NSG mice with orthotopic human PDX tumors were treated with vehicle, MRTX1133 alone, adoptive human NK cell therapy alone, or the combination of MRTX1133 and NK cells.

3) NK cell depletion study: To determine the functional contribution of NK cells to MRTX1133 efficacy, a cohort of mice with orthotopic KPC tumors was treated with an anti-NK1.1 depleting antibody alongside MRTX1133 or vehicle.

#### Types of observations and measurement techniques

1) Tumor burden and survival: Tumor growth was monitored longitudinally via ultrasound imaging and quantified by measuring tumor weight or volume at endpoint. Animal survival was tracked and analyzed using Kaplan-Meier curves.

2) Immunophenotyping: Immune cell populations in tumors, spleens, and peripheral blood were extensively profiled using multicolor flow cytometry. Immunohistochemistry (IHC) and IF were used to assess immune cell infiltration and spatial localization within tumor tissues.

#### Randomization for all in vivo animal studies

Mice were randomly assigned to the various experimental and control groups after tumor establishment. Randomization was performed using a computer-generated random number sequence to ensure unbiased allocation. Investigators were blinded to the treatment group allocation during data collection (e.g., tumor measurement and flow cytometry analysis) and subsequent analysis whenever feasible. Data collection and processing were performed on grouped samples based on their assigned treatment cohort. The animal populations from which the samples were taken are specified above (e.g., C57BL/6J mice for syngeneic models and NSG mice for PDX models).

### Mice and orthotopic tumor models

Seven- to 8-week-old wild-type C57BL/6, B6 Ptprc^a^ (common name: B6 CD45.1), and NOD-SCID (common name: NSG) mice were obtained from the Jackson Laboratory (JAX) and Laboratory Animal Center at Sun Yat-sen University. The animals were acclimated at the institution for at least 1 week before experimentation. The animal experiments were approved by the Institutional Animal Care and Use Committee of the Sun Yat-sen University and the Affiliated Provincial Hospital of Fuzhou University (IACUC-2022090301 and IACUC-2025072802).

Murine PDAC organoids were derived from pancreatic tumors in *Kras^G12D/+^; p53^R172H/+^; Pdx1-Cre^tg/+^* (KPC) mice. Human PDAC organoids were generated from PDAC tumors and genotyped (KRAS mutation status) following the protocols published by D. Tuveson’s team (Cold Spring Harbor Laboratory) at https://tuvesonlab.cshl.edu/. Orthotopic implantations of murine PDAC organoids (5000 cells, into C57BL/6 mice) and human PDAC organoids (1 × 10^6^ cells, into NSG mice) were carried out as previously described (*40*, *41*). Detailed information can be found in the Supplementary Materials.

### NK cell transfer study

For adoptive NK cell transfer studies in synergetic murine PDAC models, 5 × 10^8^ NK cells per mouse (isolated from the spleen of healthy B6 CD45.1 mice) were infused intravenously into C57BL/6 mice via the tail vein on indicated time points. MRTX1133 (Cayman Chemical, catalog no. 18165, 30 mg/kg per day, intraperitoneal injections) and recombinant murine IL-15 (PeproTech, catalog no. 210-15, 2 μg per mouse, intraperitoneal injections) were given on indicated time points. For adoptive NK cell transfer studies in human PDAC organoid–derived xenograft (PDX) model, human primary NK cells were isolated (by using EasySep cell separation kits, STEMCELL Technologies, Vancouver, BC) from PBMCs that were collected from healthy blood donors in Blood Center of the Sixth Affiliated Hospital at Sun Yat-sen University. NK cells were expanded using the ImmunoCult NK Cell Expansion Kit (STEMCELL Technologies, Vancouver, BC) for 14 days. A total of 5 × 10^8^ human NK cells per mouse were infused intravenously into NSG mice via the tail vein on indicated time points. Recombinant human IL-15 (PeproTech, catalog no. 200-15, 2 μg per mouse, intraperitoneal injections) was used to stimulate NK cells on indicated time points.

### Cell lines and culture conditions

The HPAC, LS174T, and PANC1 cell lines were obtained from National Infrastructure of Cell Line Resource (Beijing, China). The murine pancreatic cancer cell KPC was derived from a pancreatic tumor in *Kras^G12D/+^; p53^R172H/+^; Pdx1-Cre^tg/+^* (KPC) mice. Cancer cells were cultured in RPMI 1640 medium (Thermo Fisher Scientific, Richardson, TX, USA) supplemented with 10% fetal bovine serum (Gibco, CA, USA) and 1% penicillin-streptomycin (Gibco, CA, USA) and incubated at 37°C in a 5% CO_2_-humidified incubator. PDAC organoid culture was followed by the protocol previously established in our team (*41*).

### In vitro culture assays

Human primary NK cells were isolated (by using EasySep cell separation kits, STEMCELL Technologies, Vancouver, BC) from PBMCs that were collected from healthy blood donors in Blood Center of the Sixth Affiliated Hospital at Sun Yat-sen University. NK cell killing activity against prelabeled (CellTrace Violet, Thermo Fisher Scientific, catalog no. C34557) target cells were determined using the propidium iodide staining method as previously described (*42*). Detailed information can be found in the Supplementary Materials.

### scRNA-seq data analysis

Human PDAC tumors and control scRNA-seq data were obtained from a previously published study by Peng *et al.* (*43*). Uniform manifold approximation and projection (UMAP) and violin plots, along with gene expression profiles, were generated using the Tumor Immune Single-cell Hub platform (tisch.comp-genomics.org) (*44*). The raw sequencing data for this study have been deposited in the Genome Sequence Archive under project PRJCA001063 (accession number GSA: CRA001160). Murine PDAC KPC tumor scRNA-seq data from tumors treated with MRTX1133 were sourced from a prior publication by Mahadevan *et al.* (*9*). The raw sequencing data for this study have been deposited in the GEO under project PRJNA950065 (accession number GEO: GSE228502). PDX tumor scRNA-seq was used to evaluate transcriptional changes in cancer cells following MRTX1133 treatment. The raw sequencing data for this study have been deposited in the GEO under project PRJNA1130889 (accession number GEO: GSE271300). Detailed information can be found in the Supplementary Materials.

### Reverse transcription polymerase chain reaction, Western blotting, and RNA-seq analysis

The relative mRNA expression level of hICAM1, hICAM2, hULBP1, hULBP2, hMICA, hMICB, hIFNGR1, and hIFNGR2 was determined using Power-UpTM SYBRTM Green Master Mix (Thermo Fisher Scientific, catalog no. A25742) reaction system. PDAC cells and tumors were homogenized in radioimmunoprecipitation assay buffer with protease and phosphatase inhibitors to measure target proteins using Western Blotting. The RNA-seq data of HPAC xenograft were collected from a previous study (*4*). The raw sequencing data for this study have been deposited in the GEO under project PRJNA831648 (accession number GEO: GSE201412). Detailed information can be found in the Supplementary Materials.

### Flow cytometry analysis

For flow cytometry analysis, mice were administered an intraperitoneal injection of 250 μl brefeldin A (1 mg/ml) 6 hours before euthanasia. Blood, spleens, and tumors were subsequently collected and processed into single-cell suspensions following a previously established protocol (*41*). Detailed information can be found in the Supplementary Materials.

### IHC and IF staining

IHC analysis was conducted to detect p-ERK1/2, CD45.1, Ki67, ICAM-1, ULBP1, and CD11b in tumor tissue sections following a previously established protocol (*45*). For IF staining, mice were initially perfused with 30 ml of PBS to remove blood, followed by perfusion with a solution of 4% paraformaldehyde (PFA) and 0.5% methanol in PBS. Collected tumor tissues were stored in 4% PFA at 4°C for 48 hours. Subsequently, tissues were transferred to a PBS solution containing 25% sucrose until they sank and then embedded in Tissue-Tek OCT compound for the preparation of 8-μm sections for staining. Detailed information can be found in the Supplementary Materials.

### Liquid chromatography–tandem mass spectrometry analysis of 13C-U6-glucose and 13C-U16-palmitic acid flux tracing

HPAC (human) and KPC (murine) cell lines were initially cultured in standard RPMI 1640 with 10% FBS. Subsequently, cells were treated with 20 nM MRTX1133 for 24 hours. Following this incubation, the medium was replaced with either 2 mM 13C-U6-glucose medium or 0.3 mM U16-13C-palmitic acid medium (supplemented with lipid-free FBS). MRTX1133 treatment was continued for the specified duration. Polar and nonpolar metabolites were analyzed using a Waters Acquity ultra-performance liquid chromatography (UPLC) system coupled with a Xevo TQ-S mass spectrometer, adhering to an established protocol (*46*). Metabolite peak areas were integrated using Skyline (MacCoss Lab Software) and normalized to the corresponding cell counts. Further analysis, including relative quantification of peak areas, principal components analysis, heatmap analysis, and pathway enrichment analysis, was conducted using Metaboanalyst 5.0 (*47*).

### Statistical analysis

The unpaired Student’s *t* test was used to compare the two groups. A one-way analysis of variance (ANOVA) with Tukey’s multiple comparisons test was performed to compare multiple groups. Two-way ANOVA with Tukey’s test was used for multiple groups with two independent variables. Gene fragments per kilobase per million mapped fragments (FPKM) was used to reflect the gene expression levels. The |fold change| > 2 with adjusted *P* value < 0.05 was considered for further analysis. The gene set enrichment analysis was conducted using GSEA_4.0.2 and Kyoto Encyclopedia of Genes and Genomes datasets. Mouse survival comparisons were made using Kaplan-Meier curves and the Mantel-Cox log-rank test. The statistical analysis was conducted using GraphPad Prism software (GraphPad Software Inc., San Diego, USA). *P* and adjusted *P* value < 0.05 were considered significant. More detailed materials and methods can be found in the Supplementary Materials (*4*, *9*, *40*–*45*, *47*).
