# Supplementary material for: KRASG12D inhibition reprograms the tumor-induced immunosuppressive environment and enhances NK cell–mediated antitumor immunity
Source: Sci Adv. 2026 Jul 23;12(30):eaec9236. doi: 10.1126/sciadv.aec9236 (PMC13394479; doi:10.1126/sciadv.aec9236)
Supplement: Supplementary file 1 — Supplementary Materials and Methods Figs. S1 to S10 [file sciadv.aec9236_sm.pdf]

Science Advances
AAAS

Supplementary Materials for

KRAS^{G12D} inhibition reprograms the tumor-induced immunosuppressive environment and enhances NK cell–mediated antitumor immunity

Tuo Hu *et al.*

Corresponding author: Tuo Hu, hutuo3@mail.sysu.edu.cn; Jie Cao, eycaojie@scut.edu.cn; Shiyun Lu, lushiyun121739@163.com; Xueping Huang, hxuep@mail2.sysu.edu.cn; Fangqin Xue, xfq9201125105@fjmu.edu.cn; Chunbo He, chunbo.he@fzu.edu.cn

Sci. Adv. **12**, eaec9236 (2026)
DOI: 10.1126/sciadv.aec9236

This PDF file includes:

Supplementary Materials and Methods
Figs. S1 to S10

Methods and Materials

Mice and orthotopic tumor models

Seven- to Eight-week-old wild-type C57BL/6, B6 Ptprc^{a} (Common Name: B6 CD45.1), and NOD-SCID (Common Name: NSG) mice were obtained from the Jackson Laboratory (JAX) and Laboratory Animal Center at Sun Yat-sen University. The animals were acclimated at the institution for at least one week before experimentation. The animal experiments were approved by the Institutional Animal Care and Use Committee of the Sun Yat-sen University and the Affiliated Provincial Hospital of Fuzhou University.

Murine PDAC organoids were derived from pancreatic tumors in KrasG12D/+; p53R172H/+; Pdx1-Cretg/+ (KPC) mice. Human PDAC organoids were generated from PDAC tumors and genotyped (KRAS-mutation status) following the protocols published by Dr. Tuveson' team (Cold Spring Harbor Laboratory) at https://tuvesonlab.labsites.cshl.edu/protocolsreagents/.

Orthotopic implantations of murine PDAC organoids (5,000 cells, into C57BL/6 mice) and human PDAC organoids (1x106 cells, into NSG mice) were carried out as previously described(48). Briefly, before surgery, animals were anesthetized using Xylazine (5 mg/kg) and Ketamine (100 mg/kg) through intraperitoneal injections. Murine or Human PDAC organoids were suspended in Matrigel Matrix (Corning, Glendale, AZ, USA) and total of 30 ul cell suspension was injected into the pancreatic tail.

NK cell depletion in synergetic murine PDAC models was followed by the protocol previously established in our team(47). Mice received 200 μg/mouse/day anti-NK1.1 antibody (BioXcell, Clone: PK136) three times per week. Rat IgG1 (BioXcell, Cat# BE0088) was used as isotype control.

In vitro culture assays

Human primary NK cells were isolated (by using EasySep™ cell separation kits, STEMCELL Technologies, Vancouver, BC) from peripheral blood mononuclear cells (PBMC) that were collected from healthy blood donors in Blood Center of the Sixth Affiliated Hospital at Sun Yat-

sen University. NK cells were expanded using ImmunoCult™ NK Cell Expansion Kit (STEMCELL Technologies, Vancouver, BC) for 14 days. Murine NKs a were isolated from the blood or spleens using an NK cell isolation kit (Miltenyi Biotec, Cat# 130-115-818).

For the co-culture assay, NK cell killing activity against pre-labeled (CellTrace Violet, Thermo Fisher Scientific, Inc., Cat# C34557) target cells were determined using the propidium iodide staining method as previously described(49). Additionally, we assessed the NK cell activity by checking the levels of IFNγ, Granzyme B (GZMB), and CD107a after target cell stimulation for 4 hours using flow cytometer analysis.

Single-cell RNA sequencing data analysis

Human PDAC tumors and control scRNA-seq data were obtained from a previously published study by Peng et al.(50). UMAP and violin plots, along with gene expression profiles, were generated using the Tumor Immune Single-cell Hub (TISCH) platform (tisch.comp-genomics.org)(5). The raw data is accessible via the Genome Sequence Archive (GSA) under accession number CRA001160. Murine PDAC KPC tumor scRNA-seq data from tumors treated with MRTX1133, were sourced from a prior publication by Mahadevan et al.(51). Re-analysis of this dataset was performed using the Seurat R package (version 5.2.0) within R (version 4.5.0), adhering to the guidelines provided by Satija Lab and Collaborators (https://satijalab.org/seurat/articles/seurat5_integration). The raw data is available from the Gene Expression Omnibus (GEO) with accession number GSE228502.

Patient-derived xenograft (PDX) tumor scRNA-seq were used to evaluate transcriptional changes in cancer cells following MRTX1133 treatment. Samples included one vehicle-treated and one MRTX1133-treated group, each representing a pool from three individual mice. PDX tumors grown in NSG mice for 35 days and then treated with vehicle or MRTX1133 (30 mg/Kg) for 5 days. Tumor dissociation involved mincing and enzymatic digestion in a 5 mL mixture of 4 mg/mL collagenase IV and 4 mg/mL dispase (Gibco) at 37°C for 15 minutes with 250 rpm agitation. Cells were subsequently filtered twice through a 40 μm filter. Digestion was quenched with 25 mL of DMEM containing 10% FBS, followed by centrifugation at 500 g for 5 minutes. The supernatant was removed, and cells were washed with 10 mL of FACS buffer (2% BSA in

PBS) before a final centrifugation at 500 g for 5 minutes at 4°C. Dead cells were removed using the Dead Cell Removal Kit (Miltenyi Biotec, Cat.# 130-090-101) according to the manufacturer's instructions. Following an additional wash and centrifugation, cells were resuspended in 0.5 mL of FACS buffer for positive selection of CD326-positive (EpCAM+) cells using human EpCAM MicroBeads (Miltenyi Biotec, Cat. # 130-061-101), as per the manufacturer's protocol. For scRNA-seq library preparation, 5,000 EpCAM+ cells per sample were loaded onto a 10x Chromium Controller to generate Single Cell 3' Gene Expression dual-indexed libraries (10x Genomics). Downstream scRNA-seq analysis, including expression matrix processing, was performed using the Seurat R package (version 5.2.0) within R (version 4.5.0), following the established protocol by Satija Lab and Collaborators (https://satijalab.org/seurat/articles/seurat5_integration).

RT-PCR and RNA-seq Analysis

For qRT-PCR, PDAC cells and tumors were homogenized in TRIzol (Thermo Fisher) and the total RNA was isolated by RNeasy Mini Kit (QIAGEN, cat# 74104) after chloroform extraction and isopropanol precipitation following the manufacturer’s recommendations. NanoDropTM 2000/2000c was used to measure RNA concentration. The relative mRNA expression level of hICAM1 (Forward: ATGCCCAGACATCTGTGTCC, Reverse: GGGGTCTCTATGCCCAACAA), hICAM2 Forward: CGGATGAGAAGGTATTCGAGGT, Reverse: CACCCACTTCAGGCTGGTTAC), hULBP1(Forward: TAAGTCCAGACCTGAACCACA, Reverse: TCCACCACGTCTCTTAGTGTT), hULBP2 (Forward: AGCAACTGCGTGACATTCAG, Reverse: GCCATCCTATACAGTCTCCCA), hMICA (Forward: AGGGTTTCTTGCTGAGGTACA, Reverse: GGTCTCTCTGTCCCATGTCTTA), hMICB (Forward: TGGAGACTCAAGAATCGACAGT, Revers: CTGCATAGCGCGATAGTGTG),

hIFNGR1 (Forward: TCTTTGGGTCAGAGTTAAAGCCA, Reverse: TTCCATCTCGGCATACAGCAA), and hIFNGR2 (Forward: GCAGGCTTCCCAATGGATTTC, Reverse: CCCGACAGTCACATTCCGATA) were determined using Power-UpTM SYBRTM Green Master Mix (ThermoFisher, Cat # A25742) PCR reaction system. The PCR reaction was performed and analyzed on BioER Real-Time PCR System (BioER, Hangzhou, China). The

RNA-seq data of HPAC xenograft was collected from previous study(52). DEGs (differentially expressed genes) identifying between samples were further analyzed using GESA and heatmap to identify the top effected genes related to NK cell cytotoxicity pathways.

Western Blotting

PDAC cells and tumors were homogenized in RIPA buffer with protease and phosphatase inhibitors. Protein concentration was quantified using the BCA protein assay kit (Thermo Fisher Scientific). Equal amounts of protein were separated by SDS-PAGE and transferred to PVDF membranes. Following blocking with 5% nonfat dry milk in TBS-T, membranes were incubated overnight with specific primary antibodies, Anti-IFNGR1 Rabbit pAb (YT2280; Immunoway; 1:1000), Rabbit Anti-JAK (#3332; Cell Signaling Technology; 1:1000), Rabbit Anti-Phospho-JAK1 (Tyr1034/1035) (#3331; Cell Signaling Technology; 1:1000), Rabbit Anti-STAT (#9172; Cell Signaling Technology; 1:1000), Rabbit Phospho- STAT1 (Tyr701) (58D6) mAb (#9167; Cell Signaling Technology; 1:1000), Anti-ICAM1 (PT0133R) PT™ Rabbit mAb (YM8074; Immunoway; 1:2000), Anti-pan palmitoylation antibody（P49299S，Abmart Shanghai Co.,Ltd.，1:1000), and Rabbit Anti-beta ACTIN mAb (GB15003-100; Servicebio, 1:5000). Following primary antibody incubation, membranes were probed with HRP-conjugated secondary antibodies and developed using ECL reagents for visualization.

Flow cytometry analysis

For flow cytometry analysis, mice were administered an intraperitoneal injection of 250 µL Brefeldin A (1 mg/mL) six hours prior to euthanasia. Blood, spleens, and tumors were subsequently collected and processed into single-cell suspensions following a previously established protocol(47). Cells were blocked using FC anti-CD16/32 (BioLegend, Cat # 101320) and stained with LIVE/DEAD dye (ThermoFisher Scientific, Cat# L34966). Subsequently, cells were labeled with fluorochrome-conjugated antibodies specific for immune cell surface and intracellular markers The cells were subsequently stained using fluorochrome conjugated-antibodies for cell surface and intracellular markers for T cells and Myeloid cells populations (CD4, CD3, IFN-G, GATA3, RORrt, Foxp3, and CD19, CD8, LAG3, PD-1, TIGIT, IFN-G, NK1.1; CD11b, CD11c, CD80, CD86, EPCAM, PDL-1, CD163, CD206, CD86, IL10, IA/IE, iNOS2, GR1,

ARG1, IL10, Ly6C and Ly6G). Flow cytometry was performed utilizing an LSRII-Yellow/Green Flow Cytometer (BD Biosciences), and data analysis was conducted with FlowJo V10 software (TreeStar).

Immunohistochemistry (IHC)

IHC analysis was conducted to detect phosphorylated Extracellular-signal-regulated kinases 1/2 (pERK1/2), CD45.1, Ki67, ICAM-1, ULBP1, and CD11b in tumor tissue sections following a previously established protocol(53). Briefly, Formalin-fixed, paraffin-embedded (FFPE) tissue blocks were sectioned at 4 μm and mounted on positively charged slides. Sections were deparaffinized with xylene and rehydrated through a graded ethanol series. Antigen retrieval was performed by incubating sections in citrate buffer (pH 6.0) at 115°C (high pressure) for 20 minutes. Endogenous peroxidase activity was blocked with 3% hydrogen peroxide for 10 minutes. Non-specific binding was minimized by incubation with blocking solution (2.5% normal horse serum in PBS) for 30 minutes at room temperature. Sections were then incubated overnight at 4°C with primary antibodies diluted in blocking solution: Ki67 (CST Technology, Catalog No. 9027S, 1:100), pERK1/2 (CST Technology, Catalog No. 4370S, 1:100), and CD45.1 (BioLegend, Catalog No. 110702, 1:100). Following three PBS washes, sections were incubated with a horseradish peroxidase (HRP)-conjugated secondary antibody (Vector Laboratories, Burlingame, CA, USA) for 30 minutes at room temperature. Immunoreactivity was visualized using 3,3'-diaminobenzidine (DAB) chromogen (Vector Laboratories) for 5-10 minutes, followed by hematoxylin counterstaining. Sections were subsequently dehydrated, cleared, and mounted with a permanent mounting medium.

Immunofluorescent (IF) staining

For immunofluorescent staining, mice were initially perfused with 30 mL of PBS to remove blood, followed by perfusion with a solution of 4% paraformaldehyde (PFA) and 0.5% methanol in PBS. Collected tumor tissues were stored in 4% PFA at 4°C for 48 hours. Subsequently, tissues were transferred to a PBS solution containing 25% sucrose until they sank, then embedded in Tissue-Tek OCT for the preparation of 8 μm sections. The following primary antibodies were utilized for target protein detection: anti-Cleaved Caspase-3 (CC3) (CST Technology, Catalog No.

9664S, 1:100), anti-CK19 (Abcam, Cat #: ab52625, 1:200), anti-NK1.1 (BioLegend, Cat #: 108701, 1:50), and anti-CD45.1 (BioLegend, Cat #: 157602, 1:75). To assess protein co-occurrence of LAMP and IFNGR1, colocalization analysis was conducted using ImageJ 1.43m (54). For each fluorophore, a symmetrical region of interest (ROI) of consistent size was defined. Background levels were subtracted from each ROI (calculated to one standard deviation) prior to analysis. Images were decomposed into individual RGB channels. Quantitative colocalization was determined by calculating Pearson's (r) and Manders' (R) coefficients for the red and green channels using the software's embedded colocalization plugin at default settings. Results were derived from a minimum of n = 6 ROIs per pairwise comparison.

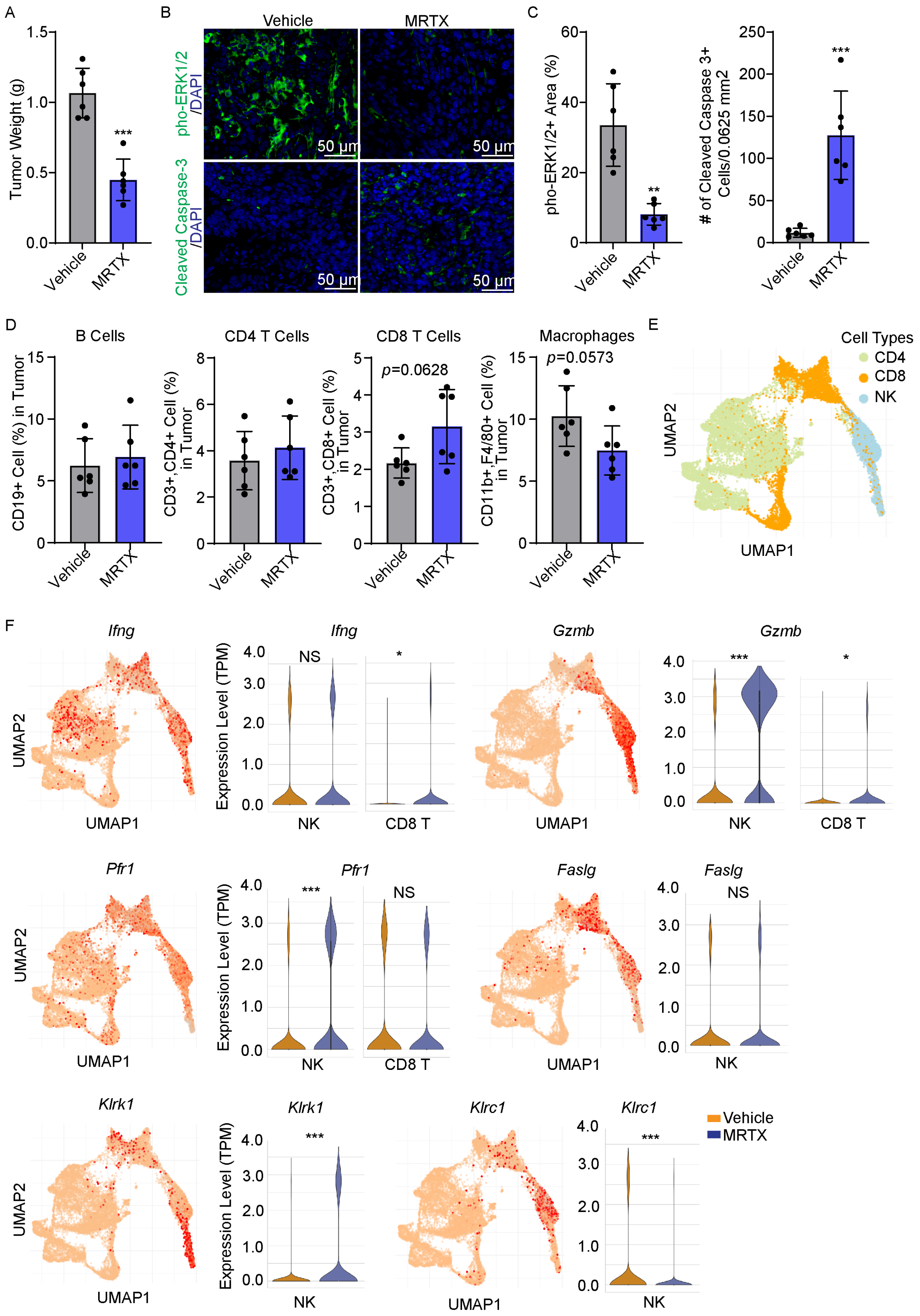

Figure S1. KRAS^{G12D} inhibition reprograms TME and enhances NK Cell activation. (A) Statistical analysis of tumor weight from orthotopic KPC tumors in C57BL/6J mice treated with vehicle or MRTX1133 for two weeks (24 days post-implantation). **(B-C)** Representative immunofluorescence images and statistical analyses of phosphorylated ERK1/2 (p-ERK1/2)

and cleaved Caspase-3 in orthotopic KPC tumors from vehicle and MRTX1133-treated groups. **(D)** Flow cytometry analysis showing the percentages of B cells, $CD4^+$ T cells, $CD8^+$ T cells and macrophages in orthotopic KPC tumors from the indicated treatment groups. **(E)** Uniform Manifold Approximation and Projection (UMAP) plot of T/NK cell populations from vehicle- or MRTX1133-treated KPC tumors, based on single-cell RNA sequencing (scRNA-seq) data (GEO: GSE228502). **(F)** UMAP and violin plots showing expression of *Gzmb*, *Ifng, Prf1*, *Fasl*, *Klrk1*(*Nkg2d*) and *Klrc1* expression in tumor infiltrating NK cells and $CD8^+$ T cells, based on scRNA-seq data. Data are expressed as Mean ± SEM. Statistical significance was determined using two-tailed Student's t-test for panel A, C, and D. *: p<0.05, **: p<0.01, and ***: p<0.001.

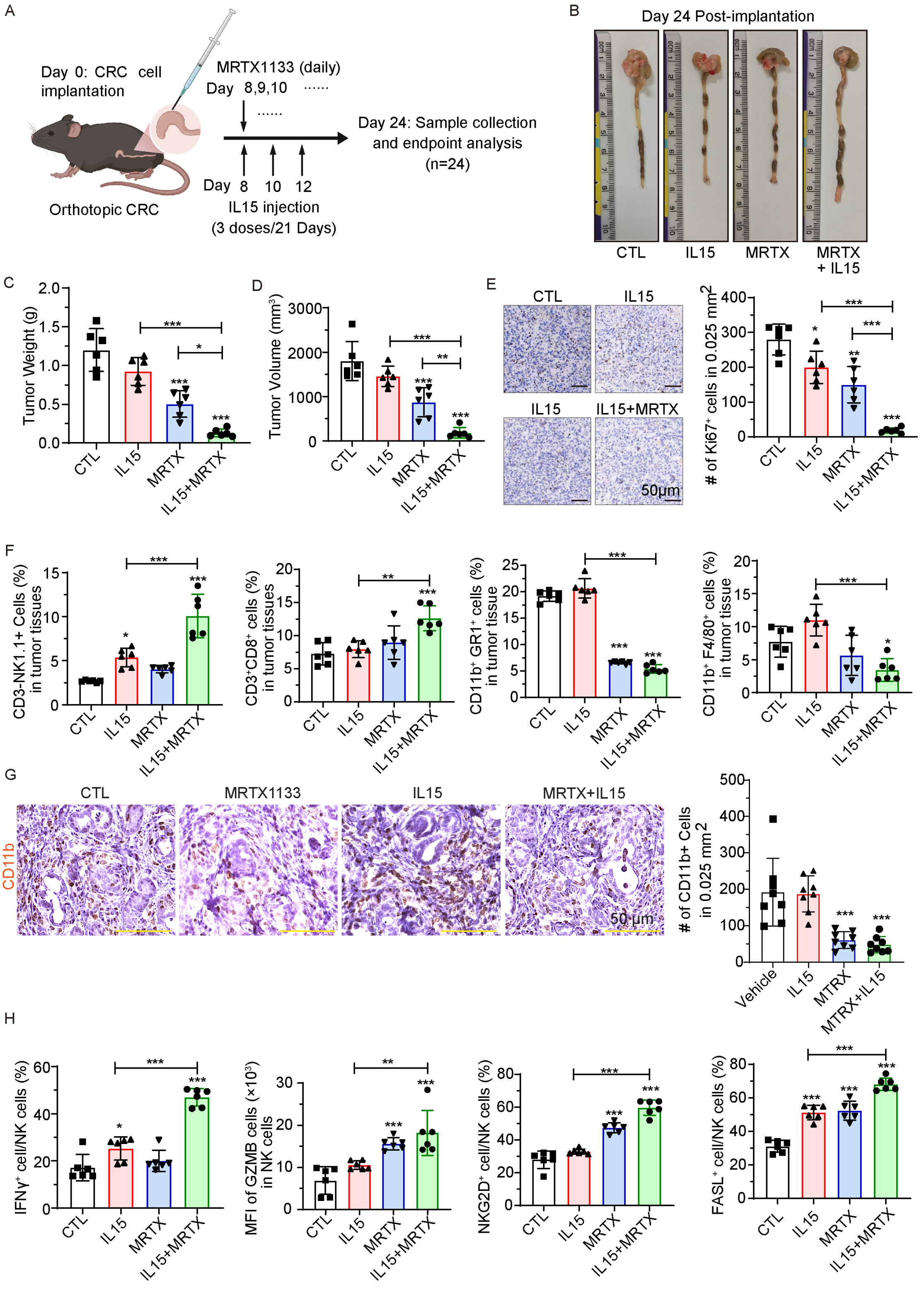

Figure S2. KRAS^{G12D} inhibitor in combination with NK cell therapy exhibits potent anti-tumor effect. (A) Schematic of the experimental design for evaluating combination therapy in an orthotopic colorectal cancer (CRC) KAC (KRAS^{G12D}, APCMin/+, Villin-Cre) tumor model. **(B)** Representative images of orthotopic KAC tumors treated with vehicle, IL-15, MRTX1133, or

MRTX1133 + IL-15 for two weeks. **(C-D)** Statistical analysis of tumor weight (C) and tumor volume (D) of KAC tumors from the indicated treatment groups. **(E)** Representative immunohistochemistry images and statistical analysis of Ki67 staining in orthotopic KAC tumors. **(F)** Flow cytometry analysis of the percentages of NK cells, CD8+ T cells, MDSCs, and macrophages in orthotopic KAC tumors. **(G)** Representative immunohistochemistry images and statistical analysis of CD11b staining in orthotopic KAC tumors. **(H)** Flow cytometry analysis of IFN-γ, GZMB, NKG2D, and FASL expression in tumor-infiltrating NK cells from the indicated groups. Data are expressed as Mean ± SEM. One-way ANOVA followed by Tukey's comparison for C, D, E, F, G, and H. *: $p<0.05$, **: $p<0.01$, and ***: $p<0.001$.

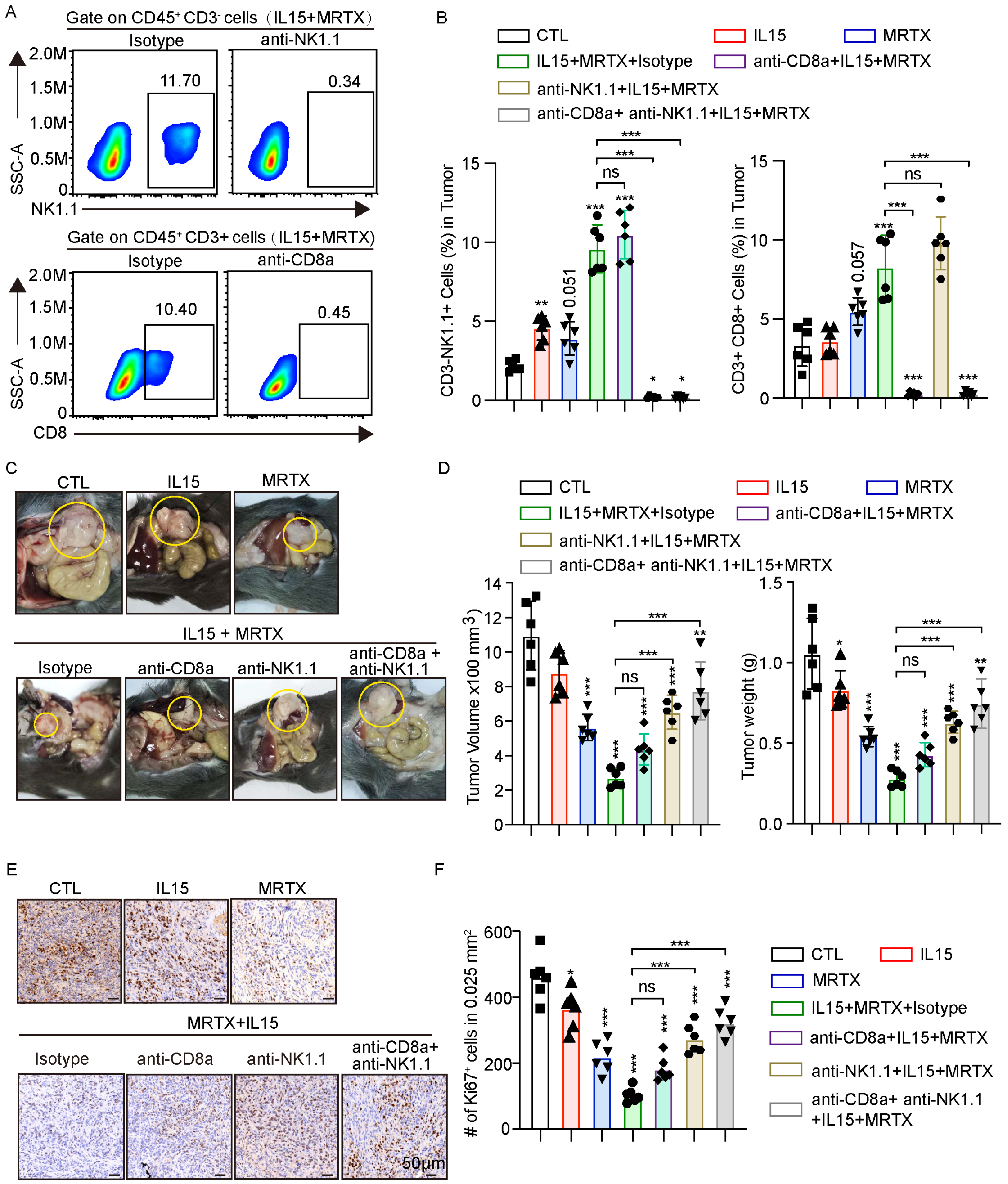

Figure S3. NK cell depletion attenuates the therapeutic efficacy of combined KRAS^{G12D} inhibition and IL-15 signaling in an orthotopic KPC model. (A–B) Representative flow cytometric plots (A) and absolute quantification (B) of infiltrating NK cells and CD8+ T cells across the specified experimental cohorts. (C–D) Assessment of tumor burden following treatment, including representative gross anatomical images of orthotopic KPC tumors (C) and statistical analysis of tumor volume and terminal weight (D). (E–F) Histological evaluation of tumor cell proliferation via Ki67 immunohistochemistry. Representative micrographs (E) and corresponding quantification of Ki67-positive nuclei (F) are provided for each treatment group.

Data are expressed as Mean ± SEM. Statistical significance was determined using one-way ANOVA followed by Tukey's comparison. Data are expressed as Mean ± SEM. Statistical significance was determined using One-way ANOVA followed by Tukey's comparison for B, D, and F. *: $p<0.05$, **: $p<0.01$, and ***: $p<0.001$.

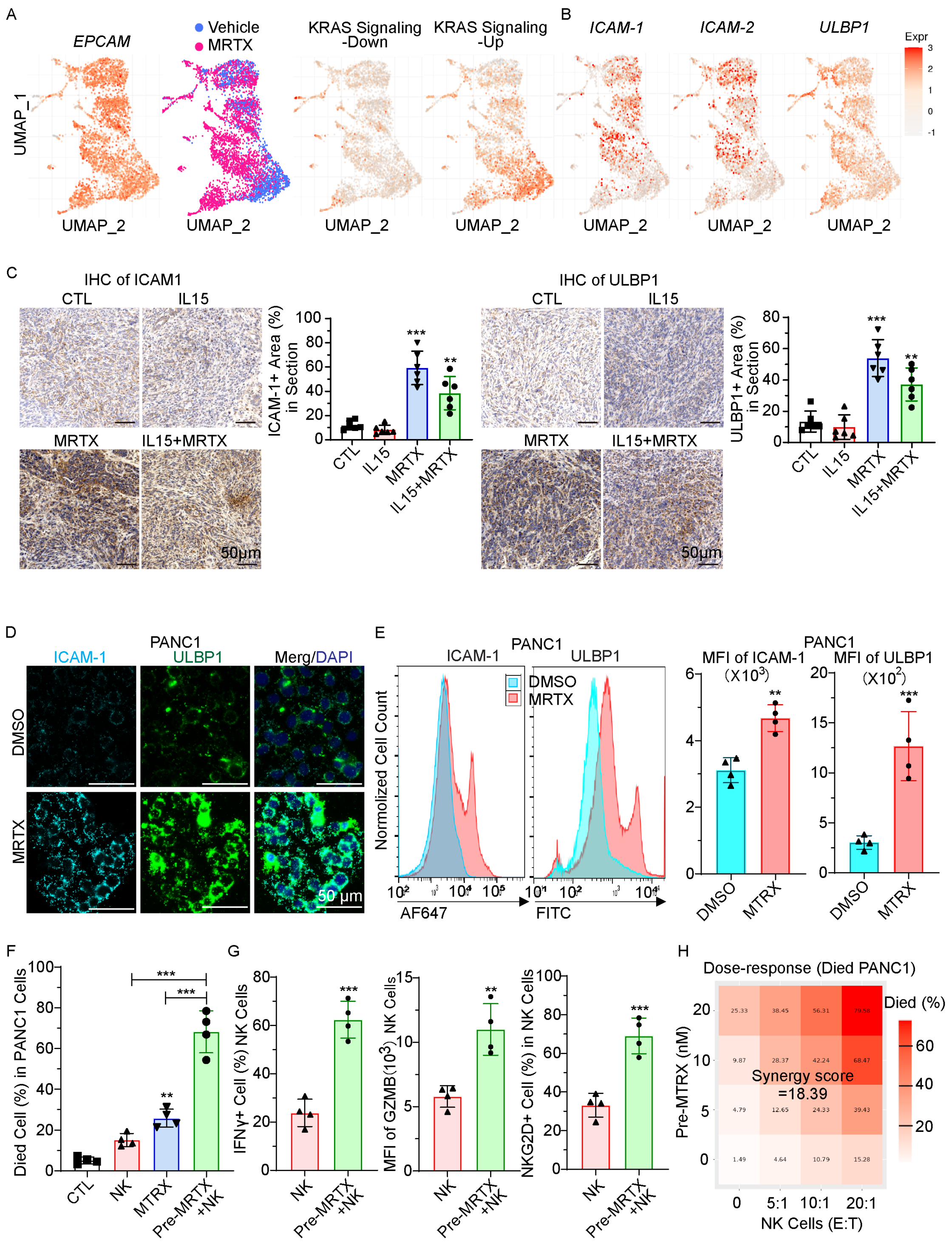

Figure S4. KRAS^{G12D} inhibition sensitizes tumor cells to NK cell killing. (A-B) UMAP plot of *EPCAM*+ PDAC cells from vehicle- or MRTX1133-treated pancreatic PDX tumors, based on scRNA-seq data for illustrating the KRAS signaling score and expression of *ICAM1/2*, *ULBP1*, and *MICA/B* in vehicle- or MRTX1133-treated pancreatic PDX tumors based on scRNA-seq data. **(C)** Representative immunohistochemistry images and statistical analysis of ICAM1 and ULBP1 staining in orthotopic KPC tumors. **(D-E)** Representative immunofluorescence staining,

flow cytometry plots, and quantification of ICAM-1 and ULBP1 in PANC1 cells treated with vehicle or MRTX1133. **(F)** Cytotoxicity assay showing the percentage of dying PANC1 cells after pre-treatment with MRTX1133 for 24 hours followed by co-culture with NK cells for 20 hours. **(G)** Expression of IFN-γ, GZMB, and NKG2D in NK cells after co-culture with vehicle- or MRTX1133-pretreated PANC1 cells. **(H)** Synergy score analysis of MRTX1133 and NK cell-mediated cytotoxicity on PANC1 cells. Data are expressed as Mean ± SEM. Statistical significance was determined using two-tailed Student's t-test for panel E and G. One-way ANOVA followed by Tukey's comparison for C and F. *: $p<0.05$, **: $p<0.01$, and ***: $p<0.001$.

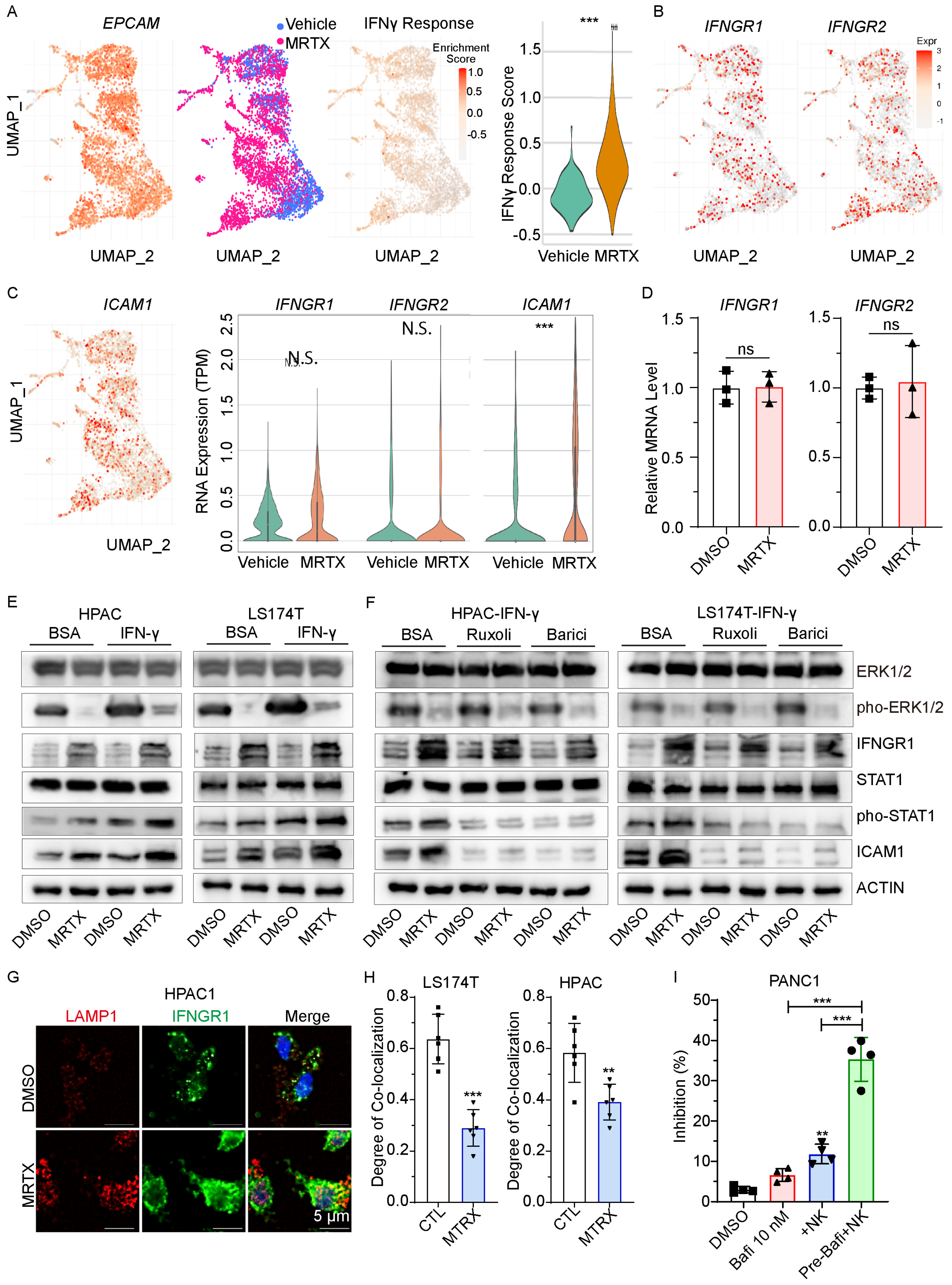

Figure S5. KRAS^{G12D} inhibition impairs IFNGR1 lysosomal degradation. (A-C) UMAP and Violin plots from scRNA-seq data illustrating the IFN-γ response signaling score and the expression of *IFNGR1*, *IFNGR2*, and *ICAM1* in *EPCAM*+ PDAC cells from vehicle- or MRTX1133-treated pancreatic PDX tumors. **(D)** Relative mRNA levels of *IFNGR1* and *IFNGR2* in HPAC cells after MRTX1133 treatment (48 hours), as determined

by qRT-PCR. (**E-F)** Immunoblot analysis of IFNGR1, ERK1/2, p-ERK1/2, STAT1, p-STAT1, ICAM1, and β-Actin in HPAC and LS174T cells after the indicated treatments. **(G)** Representative immunofluorescence images showing the effect of MRTX1133 on IFNGR1 lysosomal co-localization in HPAC cells. **(H)** Statistical analysis of colocalization of LAMP and IFNGR1 in LS174T (Figure 4D) and HPAC cells (Figure S5G). (I) Quantitative analysis showing the effect of Bafilomycin A1 (Bafi) on the sensitivity of PANC1 cells to NK cell-mediated killing. PANC1 cells were pre-incubated with Calcein AM and treated with Bafi for 24 hours before co-culturing with NK cells (target: NK=1: 10) for 20 hours. Data are expressed as Mean ± SEM. Statistical significance was determined using two-tailed Student's t-test for panel D and H. One-way ANOVA followed by Tukey's comparison for I. *: $p<0.05$, **: $p<0.01$, and ***: $p<0.001$.

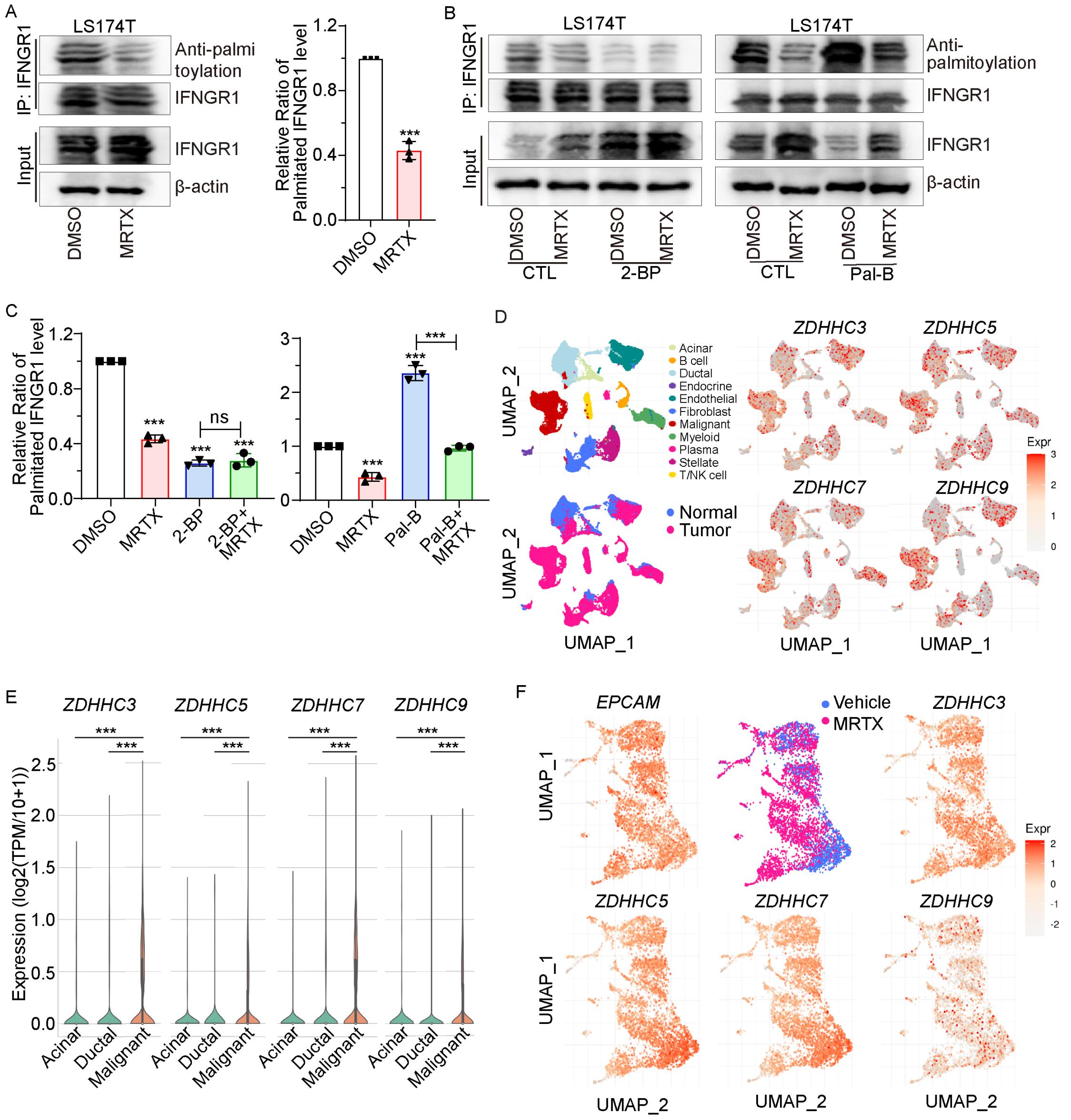

Figure S6. KRAS^{G12D} inhibition abrogates IFNGR1 palmitoylation. (A) Immunoblot analysis and quantification of total IFNGR1 (input) and palmitoylated IFNGR1 (Co-IP, relative palmitoylated-IFNGR1 ratio=anti-palmitoylation/ anti-IFNGR1 in IP part) in LS174T cells after treatment with MRTX1133 for 48 hours. **(B-C)** Immunoblot analysis and quantification of total IFNGR1 (input) and palmitoylated IFNGR1 (Co-IP) in LS174T cells after treatment with 2-BP, Pal-B, and MRTX1133 for 48 hours. **(D-E)** UMAP and violin plots showing the expression of *ZDHHC3*, *ZDHHC5*, *ZDHHC7*, and *ZDHHC9* in indicated cell types of normal pancreas and pancreatic tumors (GSA: CRA001160). **(F)** UMAP plots showing the expression of ZDHHC enzymes in *EPCAM*+ cells from vehicle- or MRTX1133-treated pancreatic PDX tumors. *: p<0.05, **: p<0.01, and ***: p<0.001.

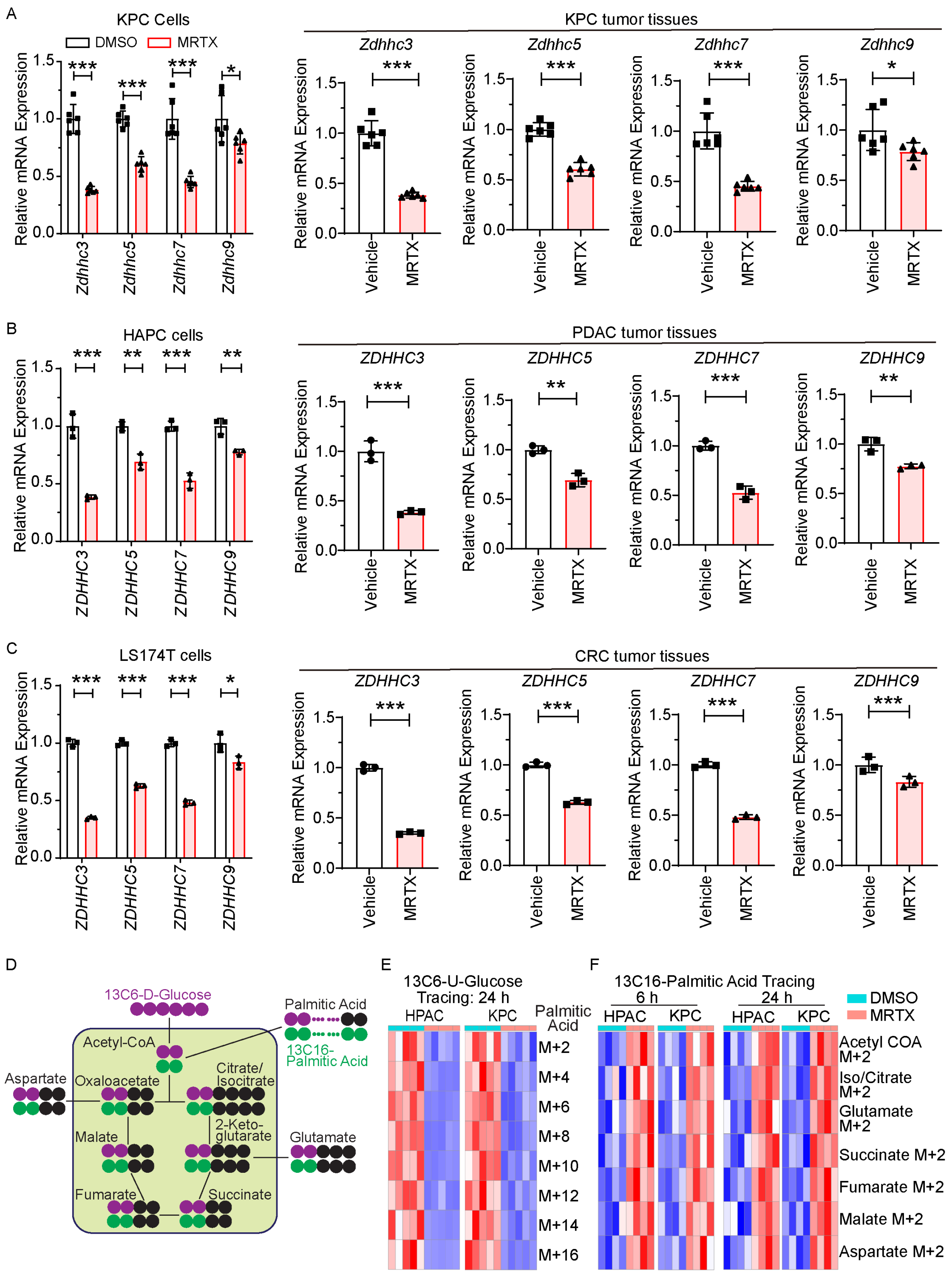

Figure S7. Pharmacological inhibition of KRAS^{G12D} suppresses palmitoyltransferase expression. (A) Relative mRNA expression levels of *Zdhhc3*, *5*, *7*, and *9* in in KPC-derived tumor organoids and orthotopic tumor tissues following treatment with vehicle or the KRASG12D inhibitor MRTX1133. **(B& C)** Quantitative analysis of *ZDHHC3*, *5*, and *9* mRNA levels in human pancreatic (HPAC) and colorectal (LS174T) cell lines, as well as in patient-

derived organoid (PDO) xenografts of pancreatic ductal adenocarcinoma (PDAC) and colorectal cancer (CRC) tissues, under vehicle or MRTX1133 treatment conditions. **(D)** Schematic illustrating metabolic flux pathways from U-13C-labeled glucose to palmitic acid synthesis (purple) and from palmitic acid into the TCA cycle (green). **(E)** Heatmap analysis illustrating the relative levels of 13C-labeled palmitic acid derived from glucose-13C in HPAC and KPC1245 cells following MRTX1133 treatment. **(F)** Heatmap analysis illustrating the relative levels of major TCA cycle metabolites labeled with palmitic acid-13C in HPAC and KPC1245 cells following MRTX1133 treatment. Data are represented as mean ± SEM. Statistical significance was determined using a two-tailed Student's t-test. Data are expressed as Mean ± SEM. Statistical significance was determined using two-tailed Student's t-test for panel A-C. *: p<0.05, **: p<0.01, and ***: p<0.001.

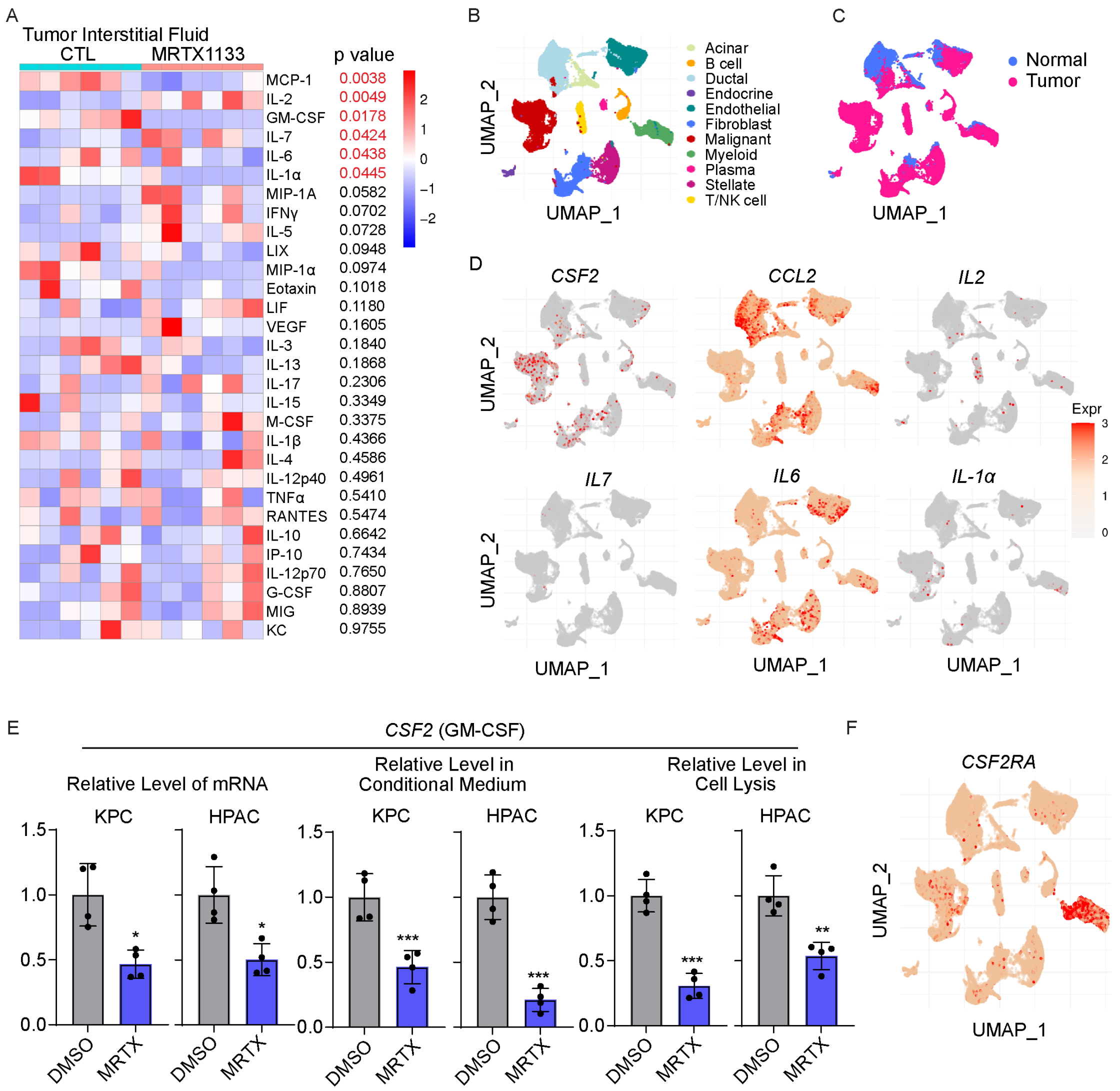

Figure S8. KRAS^{G12D} inhibition reprograms TIME and decreases cancer cell-derived GM-CSF production. (A) Cytokine array analysis of tumor interstitial fluid (TIF) from orthotopic KPC tumor-bearing mice treated with vehicle or MRTX1133 for two weeks. **(B-C)** UMAP plots of indicated cell types from normal pancreas and pancreatic tumors, based on scRNA-seq data (GSA: CRA001160). **(D)** UMAP plots showing gene expression of significantly altered cytokines across the indicated cell types. **(E)** Analysis of *CSF2* mRNA and protein levels in KPC and HPAC cells and their corresponding conditioned media following treatment with DMSO or MRTX1133 (20 nM, 48 h). **(F)** UMAP plots showing CSF2RA expression in the indicated cell types from normal pancreas and pancreatic tumors. Data are expressed as Mean ± SEM. Statistical significance was determined using two-tailed Student's t-test for panel E. *: $p<0.05$, **: $p<0.01$, and ***: $p<0.001$.

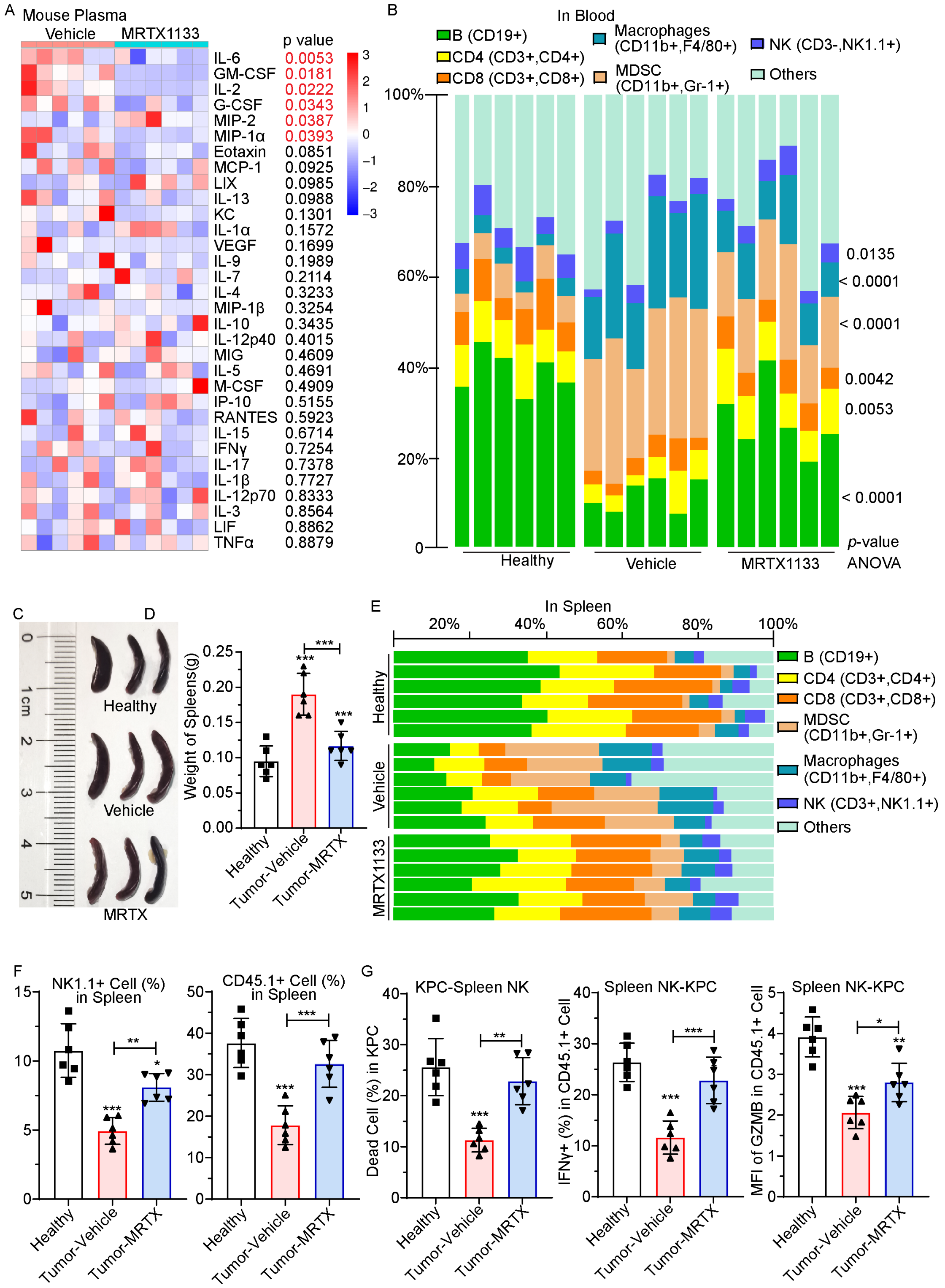

Figure S9. KRAS^{G12D} inhibition impairs tumor-induced systemically immunosuppressive effect on NK cells. (**A**) Cytokine array analysis of plasma from orthotopic KPC tumor-bearing mice treated with vehicle or MRTX1133 for two weeks. **(B)** Flow cytometry analysis of the relative abundance of indicated immune cell subsets in peripheral blood mononuclear cells

(PBMCs) from healthy or KPC tumor-bearing mice treated with vehicle or MRTX1133. **(C-D)** Representative images and weight analysis of spleens from healthy and treated KPC tumor-bearing mice. **(E)** Relative abundance of indicated immune cell subsets in spleens from healthy and treated KPC tumor-bearing mice. **(F)** Quantification of total endogenous (NK1.1+) and adoptively transferred (NK1.1+, CD45.1+) NK cells in spleens from mice described in Figure 3A. **(G)** *Ex vivo* cytotoxicity assay showing the percentage of dying KPC cells following co-culture with adoptively transferred NK cells isolated from spleens. Expression of IFN-γ and GZMB in these NK cells was also assessed. Data are expressed as Mean ± SEM. Statistical significance was determined using One-way ANOVA followed by Tukey's comparison for E and G. *: $p<0.05$, **: $p<0.01$, and ***: $p<0.001$.

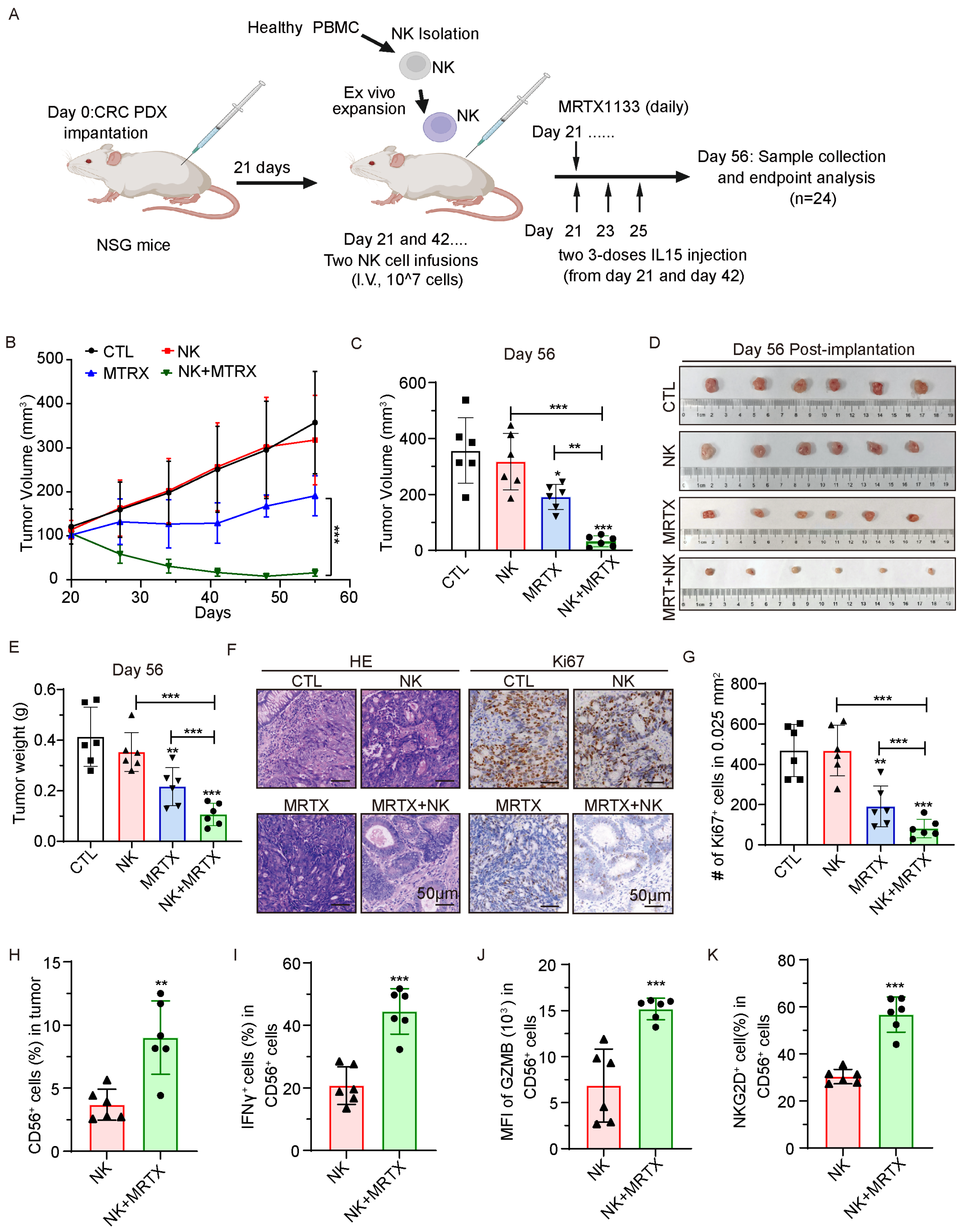

Figure S10. KRAS^{G12D} inhibition improves the anti-tumor efficacy of NK cell therapy in PDX CRC model. (A) Schematic of the experimental design for evaluating adoptive NK cell transfer in combination with MRTX1133 in an orthotopic colorectal cancer (CRC) PDX model. **(B-E)** Analysis of tumor growth curves (B), tumor volume (C), representative tumor images (D), and tumor weight (E) of orthotopic CRC tumor-bearing mice from the indicated treatment groups. **(F-G)** Representative H&E and Ki67 staining images and corresponding

quantification of Ki67-positive cells in PDX tumor tissues. **(H)** Flow cytometry analysis showing the percentage of NK cells in orthotopic PDX tumors. **(I-K)** Flow cytometry analysis of IFN-γ, GZMB, and NKG2D expression in tumor-infiltrating NK cells from the indicated orthotopic PDX tumors. Data are expressed as Mean ± SEM. Statistical significance was determined using two-tailed Student's t-test for panel H-K. One-way ANOVA followed by Tukey's comparison for C, E, and G. *: $p<0.05$, **: $p<0.01$, and ***: $p<0.001$.
